# Targeting Methionine Metabolism Reveals AMPK-SAMTOR Signaling as a Therapeutic Vulnerability in Prostate Cancer

**DOI:** 10.3390/biology14050507

**Published:** 2025-05-06

**Authors:** Serdar Arisan, Ayyuce Sever, Pinar Obakan-Yerlikaya, Elif Damla Arisan, Pinar Uysal-Onganer

**Affiliations:** 1 Department of Urology, Hamidiye Medical School, Saglik Bilimleri University, 34700 Istanbul, Türkiye; serdar.arisan@sbu.edu.tr; 2 Institute of Biotechnology, Gebze Technical University, 41400 Kocaeli, Türkiye; ayyucesever7@gmail.com; 3 Department of Molecular Biology and Genetics, Faculty of Engineering and Natural Sciences, Istanbul Medeniyet University, Uskudar, 34700 Istanbul, Türkiye; pinar.obakan@medeniyet.edu.tr; 4 Science and Advanced Technology Research Center (BILTAM), Istanbul Medeniyet University, Uskudar, 34700 Istanbul, Türkiye; 5 Cancer Mechanisms and Biomarkers Research Group, School of Life Sciences, University of Westminster, London W1W 6UW, UK

**Keywords:** mTOR, SAMTOR, methionine, prostate cancer, autophagy

## Abstract

Prostate cancer (PCa) is not only one of the most diagnosed malignancies in men but also a leading cause of cancer-related mortality globally. PCa exhibits unique metabolic dependencies, particularly on lipids and glutamine, unlike many solid tumors, rather than glycolysis. Methionine metabolism plays a crucial role in these metabolic pathways, contributing to polyamine biosynthesis, DNA methylation, and cellular signaling processes. Here, we demonstrate that methionine deprivation induces selective vulnerability in AMPK-deficient PC3 PCa cells by disrupting SAMTOR–mTOR signaling and triggering oxidative stress, lipid depletion, and autophagic responses. Through functional and proteomic analyses, we show that SAMTOR directly interacts with p-AMPK and modulates cell fate under methionine-limited conditions. Our findings establish a mechanistic link between methionine sensing and metabolic stress signaling in PCa, offering a new avenue for targeted intervention.

## Simple Summary

Targeting metabolic vulnerabilities in prostate cancer (PCa) can be challenging when its distinct metabolic profile does not align with common therapeutic strategies. Using functional and proteomic analyses, we found that while methionine metabolism is crucial for PCa cell survival, AMPK-deficient cells display heightened vulnerability under methionine-limited conditions. Despite the central role of methionine in DNA methylation, lipid metabolism, and cell signaling, its deprivation led to disrupted SAMTOR–mTOR signaling, oxidative stress, and autophagic activity. Notably, we observed that SAMTOR interacts with p-AMPK and influences cell fate decisions during nutrient stress. We suggest that exploiting methionine dependency, particularly in metabolically compromised PCa subtypes, may offer a promising avenue for targeted therapy.

## 1. Introduction

The metabolic regulation of cancer cells is significant because of the increased and highly differentiated metabolic needs compared to normal cells [1]. Food sources, mainly used by cells, cause the emergence of cancer-specific metabolites and they are formed due to the interactions of differentiated cell signaling pathways in cancer cells [2,3]. Methionine is one of the leading substances for regular metabolic cellular processes [3]. The tumor cells depend on methionine, which is well-known as an exogenous source of amino acids [4]. Methionine is an essential amino acid containing sulfur; it is vital to get it through the diet. It is used for many cell functions through a series of catabolic processes known as the methionine cycle [5,6]. The decrease in the amount of methionine in the diet shows positive results in controlling cancer cell growth and increases the therapeutic efficiency of different treatment modalities [7,8]. Previous studies indicated that limiting methionine in animal nutrition or cell culture media has metabolic effects, such as reducing the lipid signaling pathways by altering cell survival and death [3,9,10]. It was demonstrated that rats were shown to live 40% longer than the control group after being fed a diet containing 80% less methionine. Therefore, methionine deficiency increases the longevity responses of cells by triggering oxidative stress and activates autophagy [11,12,13,14].

Mammalian rapamycin target protein (mTOR) is thought to be one of the vital sensor mechanisms between autophagy and methionine amounts. mTOR is an evolutionarily conserved kinase target in the downstream signaling pathway of the PI3K/AKT signaling cascade [15,16]. mTOR is a highly conserved serine/threonine kinase that regulates cellular energy status, growth, and metabolism. Encoded by a single gene in eukaryotes, mTOR forms two distinct complexes: mTORC1, containing RAPTOR and PRAS40, and mTORC2, which includes RICTOR, mSIN1, and Protor-1/2; both complexes share mLST8 and DEPTOR [17]. mTORC1 plays a central role in amino acid sensing, particularly under nutrient-deprived conditions such as methionine restriction, which suppresses mTORC1 activity and promotes longevity-associated responses. In this context, AMPK is activated downstream and phosphorylates RAPTOR to inhibit mTORC1, further integrating metabolic stress signals. These interactions place the AMPK–mTOR axis at the heart of cancer cell metabolic regulation, particularly under oxidative and nutrient stress [17].

Genomic instability in cancer drives metabolic reprogramming, revealing methionine-dependent vulnerabilities. SAMTOR, a lysosomal SAM sensor, regulates mTORC1 by binding GATOR1 and KICSTOR to suppress its activation under methionine-deprived conditions [16]. When SAM levels are sufficient, SAMTOR dissociates from GATOR1, allowing mTORC1 activation via a mechanism that remains incompletely defined [18,19]. Methionine restriction (MetR) lowers intracellular SAM, stabilizing the SAMTOR–GATOR1 complex and inhibiting mTORC1 activity in a SAMTOR-dependent manner [16,20]. Given mTORC1’s established role in cellular aging and metabolism [21,22], SAMTOR emerges as a key regulator linking methionine availability to mTOR signaling and cancer progression.

PCa remains a significant cause of cancer-related mortality worldwide [23], with androgen receptor (AR) signaling playing a central role in tumor initiation and progression [10,23]. Beyond its role in cell proliferation, AR regulates key enzymes in one-carbon (1C) metabolism, including those involved in SAM homeostasis, the trans-sulphuration pathway, and polyamine biosynthesis [24]. AR activity alteration during disease progression and treatment can disrupt 1C metabolism and contribute to aggressive phenotypes through epigenetic reprogramming [25]. Notably, elevated levels of the methionine-derived metabolite SAM have been implicated in advanced PCa [26].

This study investigates how methionine deprivation impacts PCa cell survival by probing the SAMTOR–mTOR axis and its interaction with AMPK, a key energy sensor. We used PC3 (cancer) and PNT1A (normal) prostate epithelial cells—wild-type and AMPK-deficient—to examine how methionine restriction influences autophagy, lipid metabolism, and cell fate. SAMTOR, a negative regulator of mTORC1, exerts its function through interactions with KICSTOR and GATOR1, which are modulated by SAM availability [16,18]. We further characterized SAMTOR-associated protein networks under methionine-deprived conditions using SAMTOR-FLAG-labeled cells. These analyses provide mechanistic insights into the metabolic vulnerabilities of PCa cells and highlight the potential of targeting SAMTOR–AMPK–mTOR signaling under nutrient stress.

## 2. Materials and Methods

### 2.1. TCGA Data Analysis

The UCSC Xena database project was used to analyze SAMTOR (C7orf60) in the 497 samples from the PCa database (TCGA-PRAD, http://cancergenome.nih.gov/; accessed on 1 November 2024). The selected samples were analyzed according to Gleason score distribution in different patient data sets [27].

### 2.2. Cell Lines and Reagents

PC3 PCa wt (ATCC CRL-1435), PC3 AMPK−/− PNT1A wt (ECACC 95012614, and PNT1A AMPK−/− cell lines were cultured in Dulbecco’s modified Eagle’s medium (DMEM; GIBCO-Life Technologies, Carlsbad, CA, USA) supplemented with 10% fetal bovine serum and 1% penicillin/streptomycin and incubated in 37 °C with 5% CO_2_. AMPK−/− cell lines were generated by the transfection and puromycin selection of cells following (Addgene 73474; pX462-hPRKAA1-gRNA_A 73474 (AMPK-A)). The selected cells were recovered following 2000 μg puromycin treatment. FLAG-tagged SAMTOR transient transfection was managed by Infect or Fugene HD liposomal agents (Promega, Madison, WI, USA) (with a 1:3 or 1:6 optimization ratio) to investigate the upstream signaling pathway of mTOR that interacts with SAMTOR (Addgene 100508) [16]. Cells seeded into 6-well plates with 50% confluency were controlled for having SAMTOR expression in total protein lysate. Cell viability was investigated by morphological tests in light microscopy and the MTT cell viability test.

### 2.3. MTT Cell Viability and Cell Survival Assay

The effects of methionine starvation on PC3 PCa and PNT1A prostate epithelial cells (wt and AMPK−/−) viabilities were determined by a colorimetric MTT (3-(4,5-dimethylthiazol-2-yl)-2,5-diphenyltetrazolium bromide) assay. The cells were seeded at a density of 1 × 10^4^ per well in 96-well plates and treated with methionine-free media for 24 h. Then, 10 µL of MTT reagent (5 mg/mL in PBS, Sigma; St. Louis, MO, USA) was added to the culture medium and incubated at 37 °C for 4 h. To solubilize the formazan crystals converted from MTT by the mitochondrial enzymes, 100 μL DMSO (Sigma; St. Louis, MO, USA) was added. The absorbance of the suspension at 570 nm was measured with a microplate reader (Bio-Rad, Hercules, CA, USA). A total of 25 × 10^3^ cells per well were seeded into 12-well plates and exposed to methionine starvation in a time-dependent manner. Following incubation for 0 to 72 h, cells were counted by using an automatic cell counter from NanoEnTek (Cambridge, UK) after staining with trypan blue dye (0.4% *w/v*) [8,10].

### 2.4. Cell Cycle and Cell Death Analysis

PC3 PCa and PNT1A prostate epithelial cells (wt and AMPK−/−) were seeded into 6-well plates at 3 × 10^5^ cells/well and then incubated for cell attachment overnight. Cells were exposed to methionine starvation for 0–48 h. Following treatment, attached and non-attached cells were collected and fixed with 70% ethanol. They were washed with 1X PBS and stained with 40 μg/mL PI after incubating in the dark for 10 min. They were read and analyzed using cell flow cytometry (Accuri™ C6 Flow Cytometer, BD Biosciences, Ann Arbor, MI, USA). PC3 PCa and PNT1A prostate epithelial cells (wt and AMPK−/−) were seeded into 100 mm Petri dishes at 1 × 10^6^ cells/well and incubated overnight for cell attachment. Then cells were treated with a methionine-free medium for 24 h and 48 h. Following treatment, the cells were washed with 1× PBS, dissociated with trypsin, and centrifuged at 300× *g* for 5 min. PBS was removed from the pellet. Then, the FITC Annexin V Apoptosis Detection Kit (Biolegend, San Diego, CA, USA) was applied according to the specified protocol; the pellet was mixed with 100 μL Annexin V suspension solution (dissolved in 10 mM HEPES, 140 mM NaCl, and 2.5 mM CaCl_2_ 500 mL ddH_2_O). After adding 2 μL Annexin V and PI, samples were incubated in the dark for 15 min. The samples were read and analyzed using flow cytometry.

### 2.5. Colony Formation Assay

PC3 PCa and PNT1A prostate epithelial cells (wt and AMPK−/−) were seeded at 2.5 × 10^3^ cells/well in 6-well plates and dispersed evenly by gently shaking the dishes for 24 h. After attachment, the cells were treated with a methionine-free medium depending on the time. After 24 h, methionine-free media were removed, and the cells were allowed to form colonies in complete media for 10 days. The colonies were fixed with a solution of acetic acid and methanol (1:3) for 5 min; the supernatant was removed. Then, the cells were stained with 0.5% crystal violet for 30 min. After that, the dye was washed away with distilled water. The colonies were counted under a light microscope (SOIF, Shanghai, China).

### 2.6. Western Blot Analysis and Immunoprecipitation (IP)

PC3 PCa and PNT1A prostate epithelial cells (wt and AMPK−/−) in the presence or absence of methionine were seeded on 100 mm Petri dishes. SAMTOR-FLAG-expressing cell lines were seeded on 100 mm Petri dishes and 1 × 10^6^ cells/well, then incubated for cell attachment overnight. They were treated with a methionine-free medium and the controls with a complete medium for 24 h. Following drug treatment, cells were scraped with ice-cold 1× PBS and lysed on ice in a protein lysis solution M-PER Mammalian Protein Extraction Reagent (Thermo Scientific, Waltham, MA, USA). After the lysis procedure at +4 °C for 15 min, cell debris was removed by centrifugation for 30 min at 13,200 rpm. The Bradford protein analysis assay was performed to determine protein concentration. Total protein lysate (20–30 µg) was loaded and separated on a 10–12% SDS-PAGE and then transferred onto polyvinylidene difluoride (PVDF) membranes (Ecobiotech, Erzurum, Turkiye). The membranes were then blocked with 5% non-fat milk blocked membranes dissolved in 0.1% TBS-T (10 mM Tris-HCl and Tween 20) and incubated with appropriate primary antibodies and horseradish peroxidase (HRP)-conjugated secondary antibodies overnight at 4 °C following the addition of an enhanced chemiluminescence reagent. The antibodies used were as follows: mTOR, p-mTOR (Ser2448), ACC, FASN, ATG5, Fumarase, Lamtor, Lamtor c11/orf, LC3 A/B, and β-actin; polyclonal anti-rabbit/mouse antibodies were purchased from Cell Signaling Technology (C.S.T., Danvers, MA, USA). Each antibody was diluted in 5% non-fat milk at 1:500–1:000 concentrations—HRP-conjugated secondary anti-rabbit (1:1000). Images were taken were taken at different time points and analyzed using Image Lab Version 5.2.1.

Following the total protein extraction, for complexes with FLAG antibody-containing resin (Biolegend, San Diego, CA, USA), immunoblotting was performed after mixing the protein content at a ratio of 1:1. Results were analyzed regarding target interacting proteins for FLAG-SAMTOR targets. At this stage, the aim was to show the changes of proteins with SAMTOR binding potential that are altered by methionine starvation in wt PC3 and PNT1A cells after SAMTOR plasmid transfection. Antibodies (CST, Danvers, MA, USA), selected for this purpose involve associated signaling pathways: Rag and LAMTOR family, AMPK and ACC Antibody Sampler Kit, AMPK Subunit Antibody Sampler Kit, Fatty Acid Metabolism, and SAMTOR were identified in both cell lines [16].

### 2.7. Oil-Red O Staining and BODIPY^®^ 493/503 Staining-Fluorescence Measurement

Oil Red O staining was used to show lipid droplets, the amount of which may vary depending on the application in PCa cells. After removing old media from cells, they were washed with 1X PBS and fixed with 4% paraformaldehyde for 1 h. Then pre-warmed 0.25% Oil Red O solution (prepared in 0.5% Oil Red O propylene glycol and diluted with ddH_2_O prior to treatment) was added. The samples were kept at 60 °C for 15 min. Samples washed with 1X PBS were visualized under an inverted light microscope. BODIPY^®^ 493/503 (4,4-difluoro-1,3,5,7,8-pentamethyl-4-bora-3α,4α-diaza-s-indacene), a natural lipid stain, was used to identify the lipid droplets inside the cell. A stock concentration (1 mg/mL, with MetOH) was prepared. The cells were incubated in the dark with a medium containing the dye for 10 min at a final 10 ng/mL concentration. Samples were analyzed using a fluorescent counterflow microscope (Z.O.E., BioRad, Hercules, CA, USA).

### 2.8. Total Reactive Oxygen Species Detection

A quantity of 50 nM of LysoTracker Red (Molecular Probes, Thermo Scientific, Hemel Hempstead, United Kingdom) was applied to the cells for 30 min, and then the red lysosomal fluorescence of cells per sample was determined by fluorescence microscopy (Ex/Em: 577/590 nm, Olympus IX70; *n* = 3). Cells were seeded onto six-well plates with 3 × 10^5^ density to detect reactive oxygen species and treated with methionine-free medium for 48 h. H_2_DCFDA (Thermo Scientific) staining (5 µM) was applied to the cells for 30 min and then analyzed by fluorescence microscopy (Ex/Em: 485/535 nm, Olympus IX70; *n* = 3).

### 2.9. Data Analysis

Statistical analysis for the effect of each application was performed using GraphPad Prism version 8.0.0 (GraphPad Software, San Diego, CA, USA, www.graphpad.com) for “Bidirectional ANOVA and *t*-test results”. All the results are presented as mean ± SD.

## 3. Results

This study investigated the effects of methionine deprivation on PC3 PCa cells (wild-type and AMPK-deficient) and PNT1A prostate epithelial cells. Methionine starvation significantly reduced cell viability in PC3 cells, particularly in AMPK-deficient cells, while having less pronounced effects on PNT1A cells. Methionine deprivation induced G1 cell cycle arrest in wild-type and AMPK-deficient PC3 cells, with AMPK-deficient cells displaying heightened sensitivity. Reactive oxygen species (ROS) production increased significantly under methionine-deprived PC3 AMPK-deficient cells, accompanied by a marked reduction in mitochondrial membrane potential and lipid droplet formation. Autophagy-related markers, including LC3 lipidation, were upregulated, indicating activation of autophagic pathways. Additionally, methionine deprivation disrupted key metabolic pathways, as shown by the downregulation of fatty acid metabolism enzymes (ACC, ACLY, and FASN) and altered mTOR and Raptor signaling. Collectively, these findings highlight methionine deprivation as a potent inducer of metabolic stress, apoptosis, and autophagic signaling, particularly in PC3 AMPK-deficient PCa cells, providing insights into therapeutic vulnerabilities associated with methionine metabolism.

### 3.1. SAMTOR (C7orf60) Expression Profile Is Related to the Gleason Index Distribution

**Figure 1:**
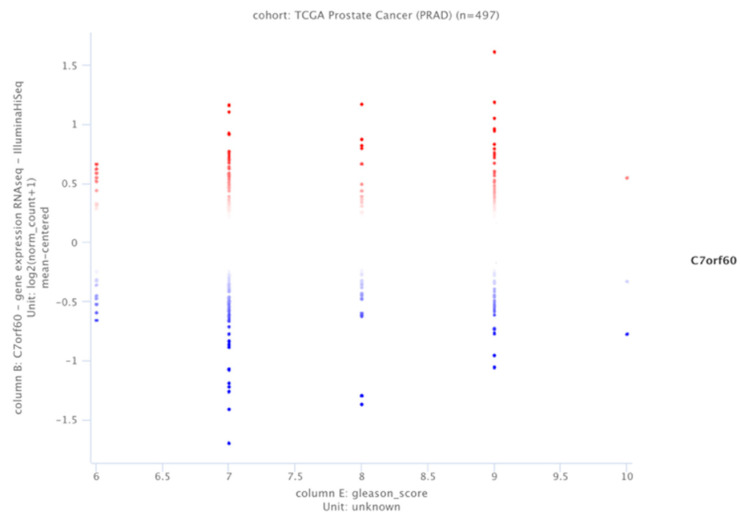
Blue dots for <8.078 thresholds for the relative expression levels (*n*: 249 patients) and red dots >8.079 (*n*: 248 patients).

According to the PRAD database, about 500 patients were analyzed for the SAMTOR expression levels and Gleason score index. The decreased SAMTOR was related to the lower Gleason score in the selected patient profile (Figure 1).

### 3.2. Methionine Starvation Led to Cell Viability Loss in Only AMPK−/− PC3 PCa Cells

**Figure 2:**
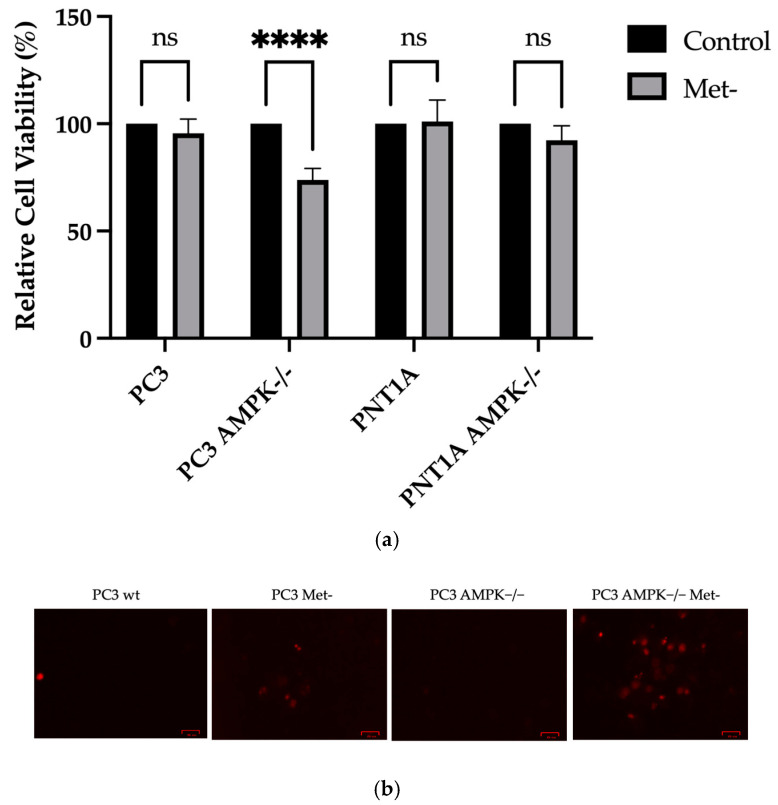
(**a**) Relative cell viability (MTT test) results with a column chart mean of at least 4 technical replicates of 3 different biological replicates’ standard deviation. Met-: Methionine deficiency for 24 h. They are presented as error values. **** *p* < 0.001, ns non-significant. untreated control (Bonferroni multiplex ANOVA analysis). (**b**) Positive staining cells from each group were used to display propidium iodide (PI) staining results. Representative images were shown from three independent replicates (*n* = 3). The scale bar is 25 μm.

Methionine deprivation (Met−) for 24 h in PC3 wt, PC3 AMPK−/−, PNT1A wt, and PNT1A AMPK−/− cells were analyzed for the cells’ viability. Considering the formation of formazan products in cells grown in a standard medium despite methionine deprivation, when mitochondria activity was examined, a significant decrease in cell viability was observed only in PC3 AMPK−/− cells (**** *p* < 0.0001 PC3 AMPK−/− Met- vs. PC3 AMPK−/− control). While no significant PI staining was observed in wt PC3 cells grown in a standard medium, it caused an increase in the number of PI-positive cells, especially in PC3 AMPK−/− cells, as in the MTT cell viability test in the case of methionine deprivation (Figure 2a). Methionine deprivation showed the potential to inhibit cell viability within 24 h more effectively in PC3 AMPK−/− cells than in PNT1A cells.

**Figure 3:**
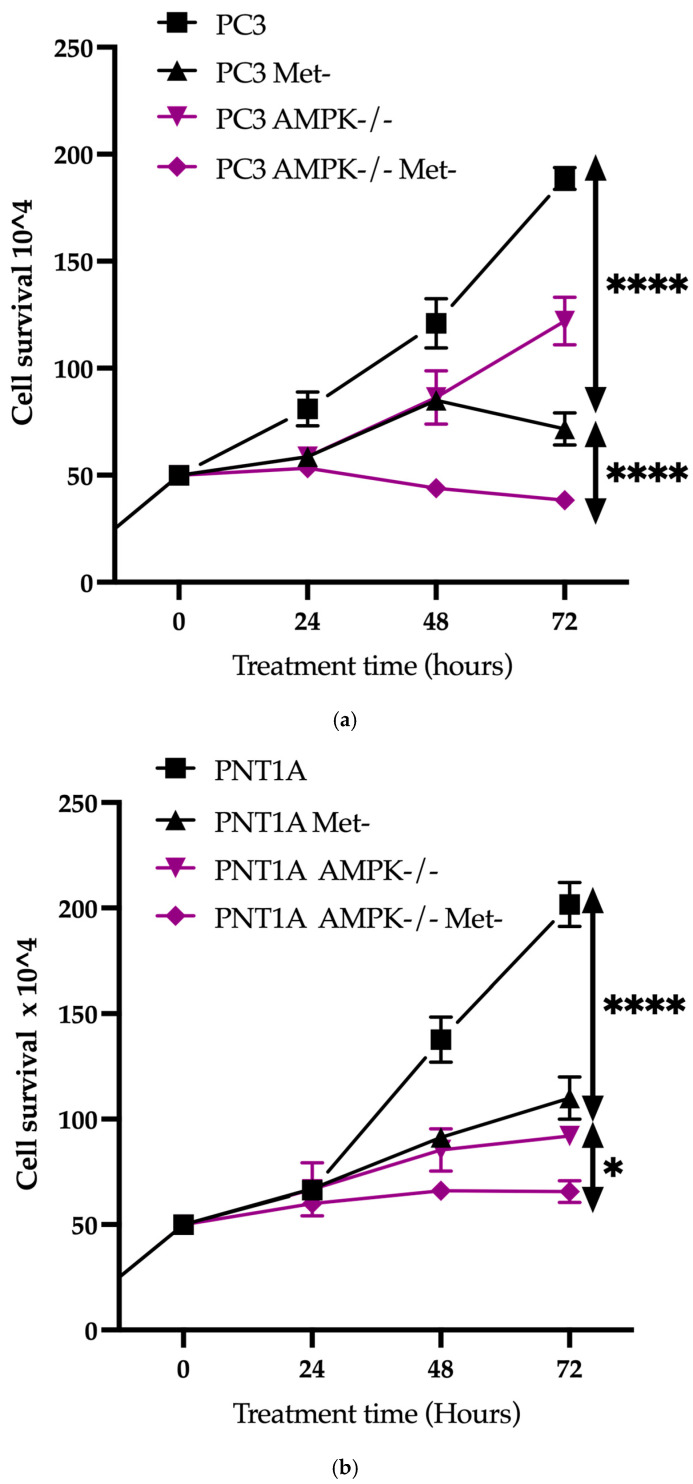
Display cell survival rates for PC3 (**a**) and PNT1A (**b**) cells. Cells were grown in a standard medium (DMEM with high glucose) and medium without methionine (Met-) between 0 and 72 h and were counted at 24 h intervals. Each 24 h count in the line graph was repeated at least three times in 2 different sets of experiments (**** *p* < 0.0001 * *p* = 0.0313, * RM one-way ANOVA analysis; Bonferroni multiple comparisons two-way ANOVA analysis) (*n* = 3).

Cell survival rate was determined in PC3 wt and AMPK−/− cells grown under methionine deprivation (Met-) or standard medium for 0–72 h. Met- conditions caused a significant decrease in cell survival (as opposed to cells grown in regular media with **** *p* < 0.001) in both PC3 wt and PC3 AMPK−/− cells. Similarly, Met- was effective in PNT1A wt cells (**** *p* < 0.001 vs. cells grown in a standard medium). However, prolonged cell proliferation in PNT1A AMPK−/− cells reduced methionine starvation (Figure 3a,b).

**Figure 4:**
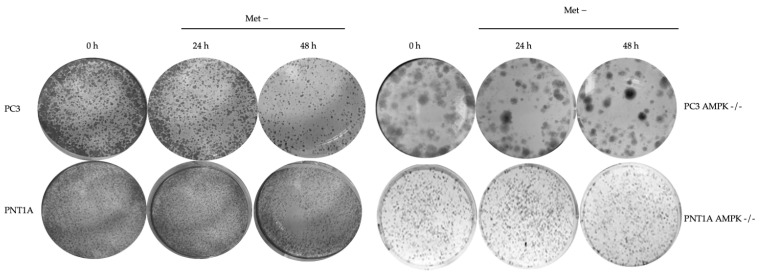
Demonstration of colony formation in PC3 AMPK−/− cells. Colony formation assay after PC3 AMPK−/− cells were exposed to methionine deprivation for 0, 24, and 48 h; PC3 AMPK−/− cells were grown for 14 days and PNT1A AMPK−/− cells for 21 days in a standard medium. The representative images are taken from triplicate experiments (*n* = 3). Then, the cells were fixed and stained with crystal violet.

The colony formation potential of the cells was examined by crystal violet staining. Large colonies in colony formation defined colony-forming characteristics of PC3 AMPK−/− cells (Figure 4). In PC3 wt and PNT1A wt cells under methionine deprivation, a significant decrease was observed in the number of colonies after 24 and 48 h of treatment. However, this decrease was higher in PC3 wt cells than in control cells. Colony diameter in PC3 AMPK−/− cells did not decrease under methionine deprivation, but a partial decrease in number was observed. Twenty-one days were kept for growth and colony formation in PNT1A AMPK−/− cells.

All cells were stained with Mitotracker dye to identify the mitochondrial membrane potential. The results showed that the mitochondrial membrane potential decreased over time in cells deprived of methionine for 48 h (Supplemental Figure S1).

**Figure 5:**
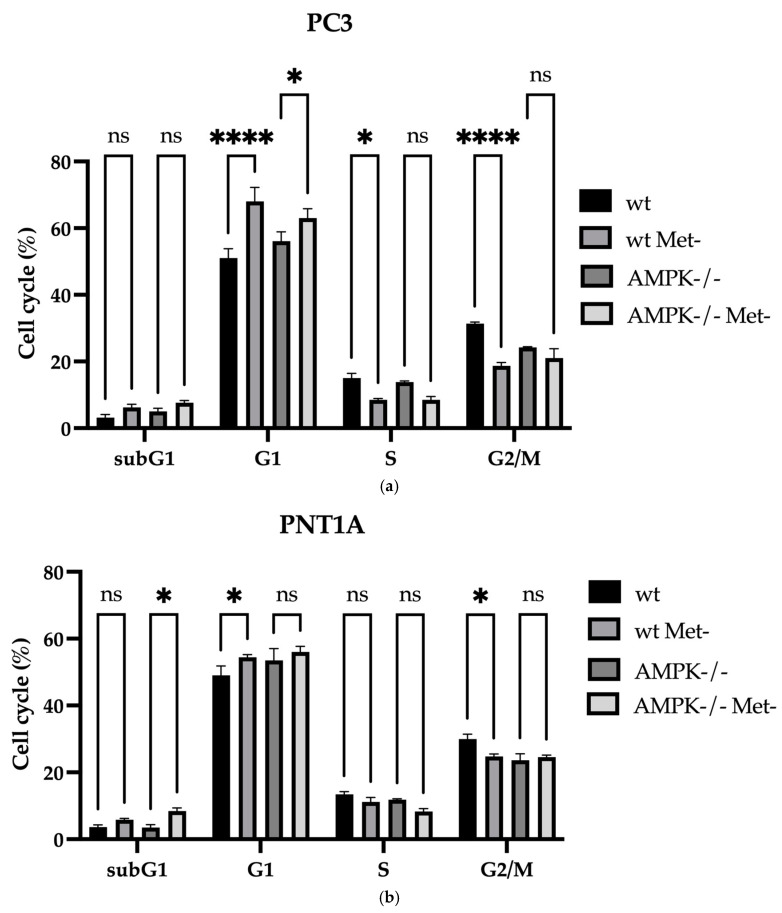

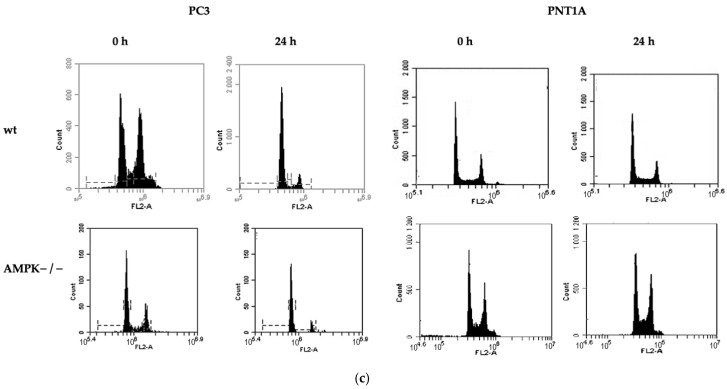
Methionine deprivation for 24 h causes cell cycle arrest in (**a**) PC3 wt, AMPK−/− and (**b**) PNT1A wt, AMPK−/− cells. The bar graphs show the mean and standard deviation of the results of three replicates plotted with error values. According to the Bonferroni multiple comparison two-way ANOVA test, in PC3 wt cells **** *p* < 0.0001, G1 phase * *p* = 0.0119, S phase * *p* = 0.0208; PNT1A cells subG1 * *p* = 0.0361; G1 * *p* = 0.0135; G2/M * *p* = 0.0192 versus control cells. (**c**) A representative image is shown for each sample (*n* = 3). Dashed lines were indicated to show the mitotic cycles. ns: non-significant.

Cell cycle analyses were performed to define the cell’s response to methionine deficiency. The readings on the cell cytometry showed that PC3 wt cells were arrested at the G1 phase under methionine deprivation conditions (Figure 5a, **** *p* < 0.0001, *n* = 3). Although this rate is more limited in PC3 AMPK−/− cells, it was determined that the cells were arrested at the G1 phase under methionine deprivation (Figure 5a, * *p* = 0.01219, *n* = 3). Consistent with this situation, methionine deprivation in wt PC3 cells for 24 h caused a decrease in cells accumulating in the S phase and G2/M phases (* *p* = 0.020 for S phase, *n* = 3; *** *p* < 0.0001 for G2/M phase, *n* = 3). Although the rate of G1 arrest in PNT1A wt cells increased at 24 h when methionine deprivation was applied, this situation was parallel with the decrease in the G2/M phase (Figure 5b, G1 * *p* = 0.0135, *n* = 3; G2/M * *p* = 0.0192, *n* = 3)—in PNT1A AMPK−/− cells, methionine deprivation caused an increase in subG1 level (Figure 5b, * *p* = 0.0292). In PC3 wt and cells, methionine deprivation increased 52.4 ± 2.1% in the G1 phase; 24 h methionine was 69.1 ± 3.8% in G1 cycle detention. Methionine starvation for 48 h resulted in 81.0 ± 5.1% of the G1 phase arrest cell population. Methionine starvation for 72 h resulted in a 91 ± 4.9% G1 phase cell population (Figure 5c).

**Figure 6:**
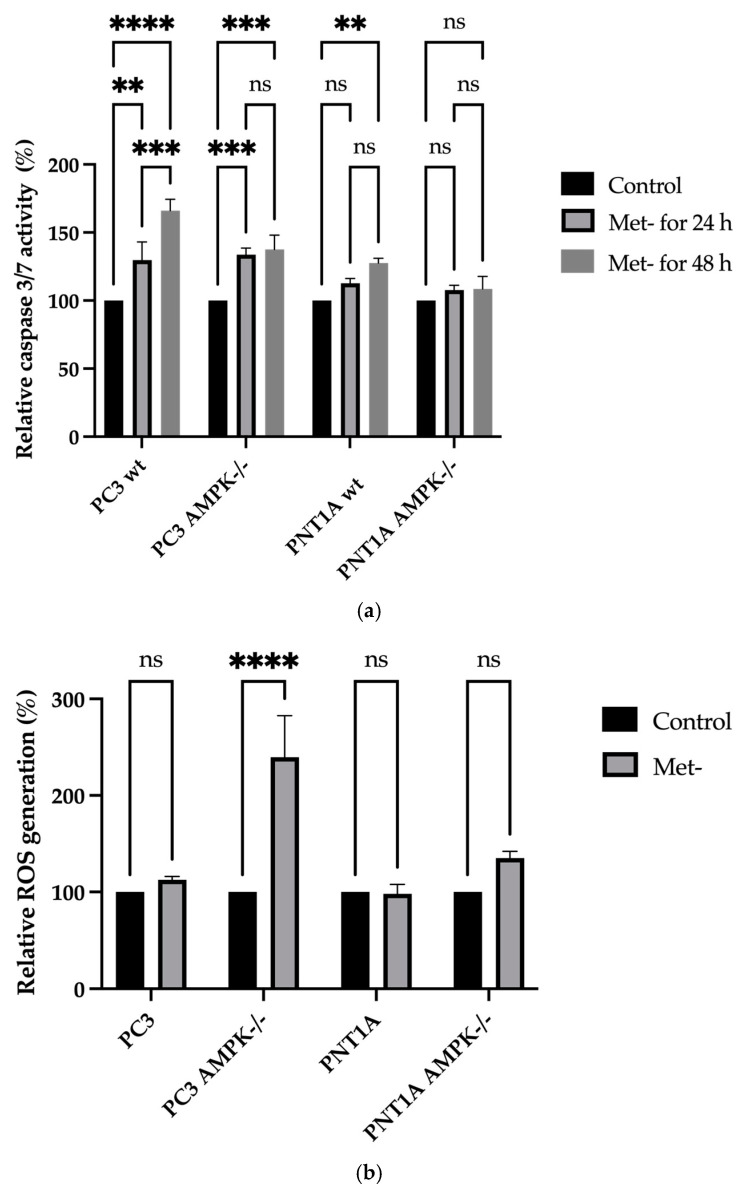
(**a**) Caspase 3/7 activity was determined following methionine deficiency in both cell lines. ** *p* = 0.0023, control vs. Met- in wt PC3 cells; **** *p* < 0.0001, *** *p* = 0.0010, control, ns non-significant. Met- in AMPK−/− PC3 cells). (**b**) Representation of ROS formation after H2DCFDA staining. In PC3 wt, AMPK−/−, PNT1A wt, and AMPK−/− cells, DCFDA dye was added to the cells after methionine starvation for 24 h (*n* = 3; **** *p* < 0.0001, control vs. Met- in AMPK−/− PC3 cells)—ns = non-significant.

Following previous findings, Caspase 3/7 activity was increased following 24 h Met- conditions in only PC3 wt and AMPK−/− PCa cells (Figure 6a, ** *p* = 0.0023, control vs. Met- in wt PC3 cells; *** *p* = 0.0010, control vs. Met- in AMPK^−/−^ PC3 cells). We did not determine any significant alteration in wt and AMPK−/− PNT1A prostate epithelial cells. In a similar trend, we found that Met deficiency increased caspase activity. The Met deficiency led to increased caspase activity compared to 24 h and control treatments (*** *p* = 0.0004 vs. 24 h treatment, ** *p* < 0.0021 vs. control in PC3 wt cells). The deletion of AMPK was protective compared to Met deficiency at both 24 h and 48 h in both cell lines (Figure 6a).

As part of the study, the survival-inhibiting effects of methionine deprivation on cell fate were demonstrated in PC3 wt and AMPK−/− cells. These cells can respond to methionine deprivation earlier than PNT1A prostate epithelial cells. Therefore, methionine starvation may be considered a stress inducer with the potential to alter cellular responses in PC3 wt and AMPK−/− deficient cells. In this context, the formation of ROS, which can be concrete proof of the stress occurring in the cells, has been determined. Met- conditions dramatically increased ROS generation in PC3 AMPK−/− cells. According to the relative expressed ROS formation values, ROS formation in AMPK-deficient cells increased approximately 2.2-fold after 24 h (Figure 6b, **** *p* < 0.0001, control vs. Met- in AMPK−/− PC3 cells).

**Figure 7:**
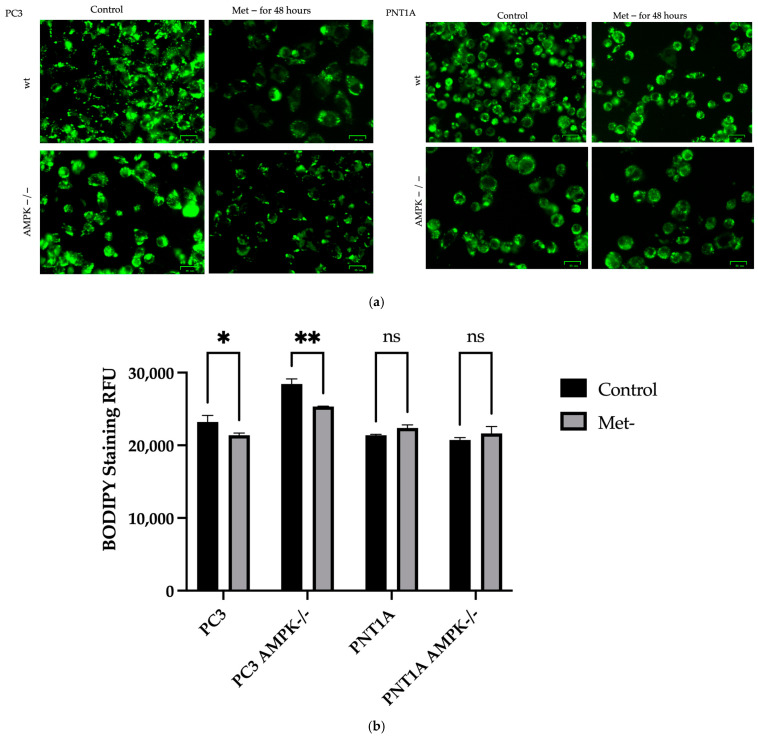
Lipid droplet determination in both cell lines following methionine deprivation conditions. (**a**) Fluorescence images are shown for the representative images from each sample following staining with BODIPY to detect natural lipids in the cells. Ex: 505 nm, Em: 515 nm readings were performed with a fluorescent reader (**b**) in cells stained with BODIPY for cytoplasmic lipid droplets. The bar graph is drawn for the mean ± standard deviation of three replicate sets of * *p* = 0.0467, ** *p* = 0.0023 control versus methionine-deficient cells (Sidak multi-way comparison two-way ANOVA test) (*n* = 3). Scale bar 25 µm.

We determined the cellular levels of neutral lipids in the cells via BODIPY and Oil Red O staining. According to significant changes in ROS generation following methionine deficiency, we hypothesized that these alterations cause a dramatic shift in lipid metabolism related to modified metabolism, especially in cancer cells. The increased ROS associated with stress, especially in PC3 wt and AMPK−/− cells, caused a significant decrease in lipid droplets (Figure 7a and Supplemental Figure S2). The reduction in cytoplasmic lipid droplets shown in detail in Figure 7b in PC3 wt and AMPK−/− cells under methionine deprivation shows that methionine starvation is vital in cellular energy regulation.

**Figure 8:**
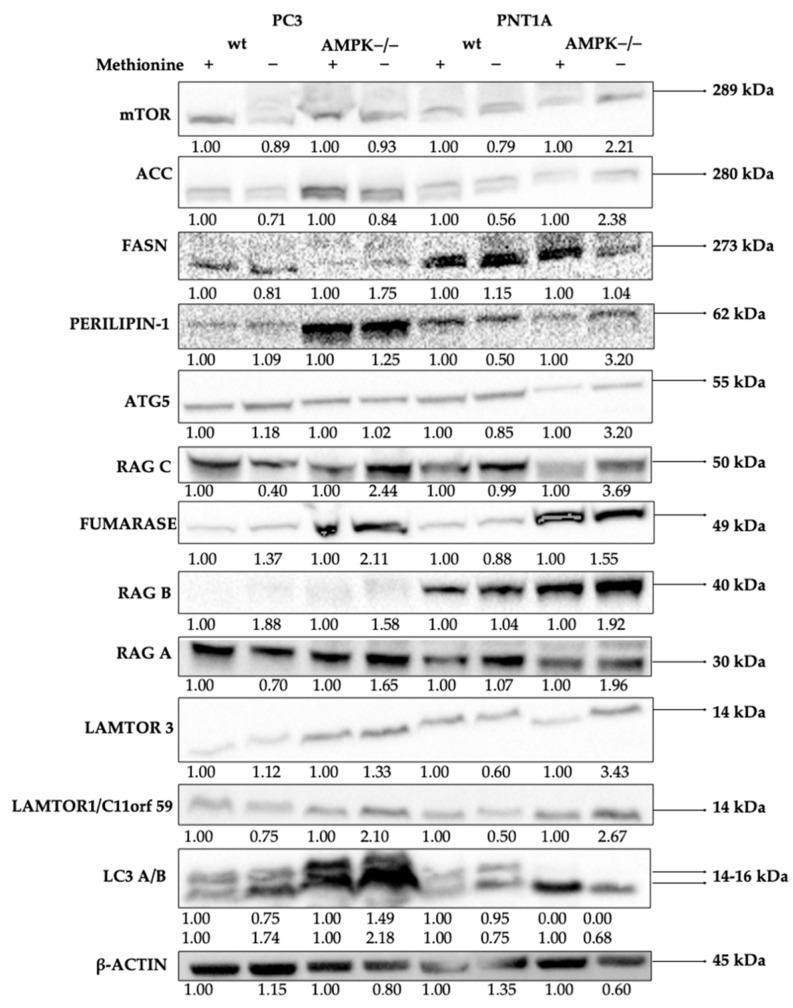
Representation of mTOR-autophagy and lipogenesis-related signaling pathways in the AMPK sub-signaling pathway in PC3 wt and AMPK−/− cells following methionine deprivation for 24 h. A quantity of 30 µg of protein was run on the gel for each sample. β-actin was included as a loading control (*n* = 3) (Supplementary Figure S4).

To investigate the decision-making mechanisms between cell survival and death under methionine deprivation, we first examined fatty acid and lipid metabolism targets in PC3 wild-type (wt) and AMPK knockout (AMPK−/−) cells. Specifically, we analyzed the expression levels of acetyl-CoA carboxylase (ACC), ATP citrate lyase (ACLY), adiponectin, CCAAT/enhancer-binding protein alpha (C/EBPα), fatty acid synthase (FASN), perilipin, and fumarase after 24 h of methionine deprivation (Figure 8 and Supplementary Figure S4). Methionine deprivation reduced ACC levels in both PC3 wt and AMPK−/− cells compared to controls. ACLY was notably downregulated, particularly in PC3 wt cells. Similarly, adiponectin expression decreased under methionine-deprived PC3 wt cells, while AMPK−/− cells showed a significant reduction as well. Interestingly, methionine supplementation led to an increase in adiponectin levels in AMPK−/− cells.

C/EBPα expression increased in PC3 wt cells following methionine deprivation, but no similar change was observed in AMPK−/− cells. FASN expression changes paralleled ACC patterns in both cell lines, while variations in perilipin expression correlated with C/EBPα levels. Fumarase expression decreased under methionine-deprived PC3 wt cells and was absent in AMPK−/− cells; however, methionine supplementation restored fumarase expression.

These results suggest that methionine deprivation can inhibit key molecular pathways associated with lipogenesis by reducing the expression of FASN, ACC, and related proteins. Importantly, the changes observed in AMPK−/− cells were similar to those in wt cells, implying that methionine deprivation independently suppresses fatty acid synthesis. Additionally, the regulation of adiponectin, an upstream activator of AMPK, was particularly notable under methionine deprivation.

ACLY, a critical enzyme in de novo lipogenesis that catalyzes the conversion of cytosolic citrate to acetyl-CoA, showed inverse changes relative to ACC under methionine deprivation. The known interactions between ACLY/AMPK and ACLY/ACC signaling in cancer cells highlight the metabolic shifts induced by methionine restriction. Furthermore, diminished AMPK expression was associated with increased autophagy markers under methionine-deficient conditions (Supplemental Figure S3). Elevated adiponectin levels in AMPK−/− cells further suggest alterations in lipid regulatory pathways.

To investigate upstream signaling changes, we evaluated mTOR pathway components in PC3 wt and AMPK−/− cells after 24 h of methionine starvation. Methionine deprivation led to increased mTOR protein expression but decreased Raptor expression in PC3 wt cells, suggesting a reduction in downstream mTOR signaling. This was confirmed by the decreased phosphorylation of p70S6K at Thr389. Similar patterns were observed in AMPK−/− cells, where AMPK deficiency further suppressed p70S6K Thr389 activity. Notably, p-4EBP Thr37/46 levels were elevated only in AMPK−/− cells and decreased under methionine deprivation, indicating excessive baseline mTOR activity in the absence of AMPK.

LAMTOR3 expression increased in both wt and AMPK−/− cells under methionine deprivation, with a stronger response in AMPK−/− cells. Conversely, LAMTOR1 expression decreased in both cell types. Increases in RagC and RagD levels, along with LAMTOR3 upregulation, suggest that these components may participate in a compensatory signaling response to methionine deprivation. RagA expression was significantly reduced in wt and AMPK−/− cells under methionine deprivation.

Taken together, these findings suggest that studying LAMTOR3 and RagA signaling in AMPK−/− PC3 cells could provide deeper insights into the methionine response mechanisms and identify new therapeutic targets.

**Figure 9:**
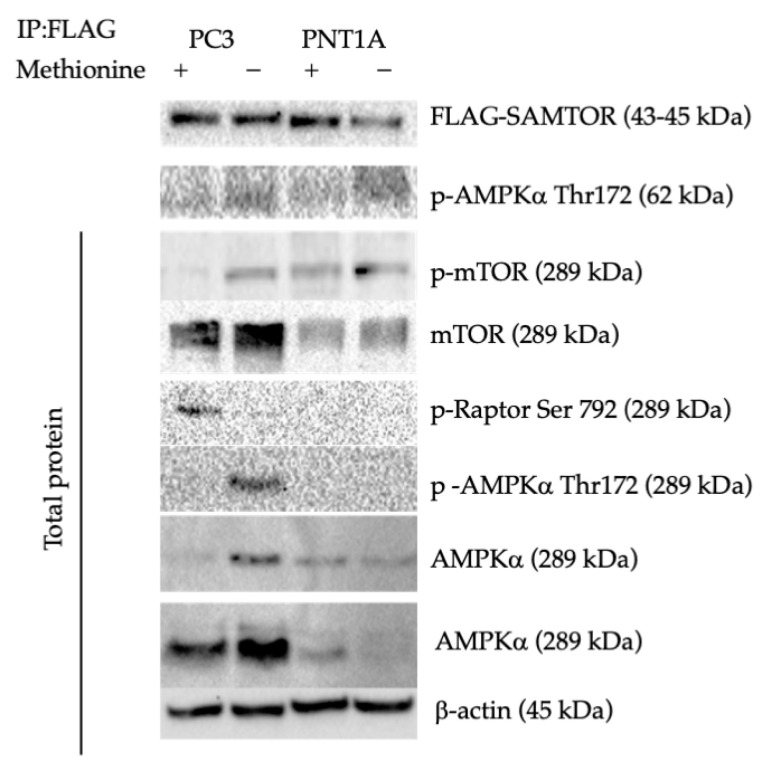
The interaction of FLAG-labeled SAMTOR protein obtained by immunoprecipitation with p-AMPK Thr172 was demonstrated in the presence (+) and absence (−) of methionine. At the same time, AMPKα, LC3B, mTOR, p-mTOR Ser2448, p-Raptor (Ser792), and p-AMPK Thr172 expression were determined in PC3 and PNT1A cells with FLAG-labeled SAMTOR expression according to total protein amounts. β-actin was included as a loading control (*n* = 3) (Supplementary Figure S4).

To identify the molecular mechanism between mTOR-AMPK and related proteins, we investigated the effect of methionine deprivation on p-AMPK by modeling PC3 SAMTOR-FLAG-expressing cells. FLAG-labeled proteins were collected by immunoprecipitation in PC3 and PNT1A cells with SAMTOR-FLAG-labeled protein expression. Total AMPK, p-mTOR, mTOR, p-Raptor, and LC3 targets were examined in PC3 and PNT1A cells with FLAG-labeled SAMTOR expression (Figure 8 and Supplementary Figure S4). In immunoprecipitated proteins, p-AMPK Thr172 interacted with FLAG-labeled SAMTOR in PC3 cells (Figure 9 and Supplementary Figure S4). Deprivation of methionine in PNT1A cells in FLAG-labeled immunoprecipitated proteins caused a decrease in the amount of FLAG-labeled SAMTOR.

The increase in AMPK was noted in PC3 wt cells with SAMTOR-FLAG-labeled protein expression under methionine deprivation. It was not detected in PNT1A cells, and methionine deprivation did not cause an increase in AMPK. An increase in the LC3B-I band was observed under methionine deficiency in PC3 cells with SAMTOR-FLAG expression at the LC3B target but not in PNT1A cells. Like LC3, mTOR protein expression was increased in PC3 SAMTOR-expressing cells under methionine deprivation.

## 4. Discussion

In this study, we elucidated the role of AMPK in PCa cell responses to methionine deprivation, focusing on its interplay with SAMTOR and mTORC1 signaling, and downstream effects on cell fate, autophagy, and lipid metabolism [28,29,30,31]. Using wild-type (wt) and AMPK-deficient (AMPK−/−) PC3 and PNT1A cell models, we demonstrated that methionine restriction (MetR) disrupts cellular homeostasis and selectively impairs survival in AMPK-deficient cancer cells. Our findings provide new insights into the metabolic dependencies of PCa cells and highlight the importance of the SAMTOR–AMPK–mTOR axis in mediating stress adaptation.

Methionine, an essential amino acid, plays a critical role in protein synthesis and one-carbon metabolism through its derivative S-adenosylmethionine (SAM), which is essential for methylation reactions, polyamine synthesis, and redox balance [4,16,18,24,26,32,33,34,35]. Cancer cells, including PCa cells, exhibit a high dependency on exogenous methionine, a metabolic vulnerability termed methionine dependence or the Hoffman effect. Unlike non-malignant cells, which can utilize homocysteine to maintain proliferation under methionine-limited conditions, most cancer cells fail to adapt and undergo cell cycle arrest or apoptosis [34,36]. Our study confirmed this phenotype in PC3 cells, particularly under AMPK-deficient conditions, where methionine starvation resulted in G1 cell cycle arrest, diminished mitochondrial membrane potential, and increased caspase-3/7 activity.

We further explored the role of SAMTOR, a lysosome-associated SAM sensor that negatively regulates mTORC1 activity by interacting with GATOR1 and KICSTOR complexes under low SAM conditions [16,18]. Overexpression of SAMTOR in PC3 and PNT1A cells modulated AMPK activity and attenuated mTORC1 signaling, as evidenced by increased p-AMPK (Thr172) and decreased mTOR activation. Interestingly, this regulation appeared context-dependent, as SAMTOR overexpression was associated with elevated p-mTOR (Ser2448) and p-Raptor (Ser792) in specific settings, suggesting feedback regulation and potential compensation by alternative kinases. These findings suggest that SAMTOR acts as a molecular switch, integrating methionine availability with energy and nutrient sensing pathways.

In the context of amino acid starvation, AMPK serves as a central regulator by sensing low ATP/AMP ratios and phosphorylating downstream targets such as RAPTOR to inhibit mTORC1 and promote autophagy [17,24,25,31,37]. Our data indicate that methionine deprivation triggers AMPK activation in a time-dependent manner, promoting LC3B lipidation and autophagy in PC3 cells. SAMTOR-mediated amplification of AMPK signaling under MetR further underscores its role in enhancing the metabolic stress response.

At the lysosomal interface, the v-ATPase–Ragulator–AXIN/LKB1 complex plays a critical role in the activation of both AMPK and mTORC1, acting as a metabolic checkpoint [17,38,39]. Our immunoprecipitation assays identified p-AMPK as a SAMTOR-binding partner, supporting the hypothesis that SAMTOR may coordinate AMPK activation through direct or indirect interactions with lysosomal scaffolding complexes. In parallel, the Rag GTPase family, essential for mTORC1 recruitment to lysosomes, showed reduced expression (RagC/D) under methionine-deprived cells, especially in AMPK−/− PC3 models, suggesting that Rag-mediated signaling is attenuated under nutrient stress and further compromised by AMPK loss.

The differential regulation of mTORC1 by amino acid sensors such as Sestrin2 (leucine), CASTOR1 (arginine), and SAMTOR (methionine) underscores the complexity of nutrient sensing in cancer [17,28,29,30,40]. Our study expands this landscape by demonstrating that SAMTOR regulates mTORC1 via SAM binding and intersects with AMPK-mediated metabolic control. The dual regulation of Raptor phosphorylation, both inhibitory (via AMPK) and potentially activating (via unknown kinases), in SAMTOR-overexpressing cells highlights the plasticity of this pathway under metabolic stress [41,42,43,44,45,46].

Cancer cells exhibit altered methyl group metabolism and upregulation of amino acid transporters such as SLC6A14, facilitating increased methionine uptake [6,24,41,42,43,44,45,46,47,48,49,50]. Our findings align with the growing recognition that targeting amino acid transporters or mimicking amino acid deprivation through metabolic interventions may sensitize cancer cells to therapeutic agents. Indeed, synergy between methionine restriction and chemotherapeutic agents like doxorubicin has been reported in sarcoma models [32,35,51,52]. The heightened sensitivity of AMPK-deficient PC3 cells to MetR observed in our study supports the potential utility of AMPK status as a biomarker for metabolic therapy responsiveness.

Additionally, the observed downregulation of fumarase in AMPK-deficient cells under MetR links between TCA cycle activity and energy stress signaling. Fumarase deficiency has been implicated in impaired AMPK activation and metabolic reprogramming [53,54], suggesting that loss of mitochondrial function may further exacerbate methionine stress in aggressive PCa phenotypes.

Collectively, our current data establish a mechanistic framework in which methionine deprivation disrupts SAMTOR–mTORC1 signaling while enhancing AMPK activation, leading to impaired survival specifically in AMPK-deficient PCa cells. This study reveals a unique metabolic vulnerability in PCa and supports the rationale for developing precision oncology strategies that target the SAMTOR and AMPK pathways to exploit methionine dependence in aggressive tumors.

However, the conclusions are currently limited by the lack of in vivo validation, which is critical to confirming the physiological relevance and therapeutic potential of these mechanisms in a more complex biological environment. Moreover, since the findings are based solely on PCa models, it remains unclear whether similar dependencies exist across other cancer types, highlighting the need for broader investigations to determine the generalizability of this therapeutic approach.

## 5. Conclusions

Our study reveals that methionine deprivation selectively impairs AMPK-deficient PCa cells by activating SAMTOR-mediated metabolic stress responses. The differential sensitivity between cancerous and non-malignant prostate cells highlights a novel therapeutic window that warrants further investigation in vivo. Targeting the SAMTOR–AMPK–mTOR axis may represent a promising strategy to exploit metabolic vulnerabilities in aggressive PCa.

## Data Availability

The data presented in this study are available on request from the corresponding authors.

## Supplementary Materials

The following supporting information can be downloaded at: https://www.mdpi.com/article/10.3390/biology14050507/s1, Figure S1: Mitochondrial membrane potential decreases following 48-h methionine deprivation, assessed by Mitotracker staining and fluorescence analysis; Figure S2: Visualization of lipid droplet accumulation in PC3 and PNT1A wild-type and AMPK−/− cells by Oil Red O staining; Figure S3: Impact of methionine deprivation on autophagy-related signaling within the AMPK pathway in PC3 wild-type and AMPK−/− cells; Figure S4: Original WB images of Figure 8 and Figure 9.
