# Supplementary material for: Targeting Methionine Metabolism Reveals AMPK-SAMTOR Signaling as a Therapeutic Vulnerability in Prostate Cancer
Source: Biology (Basel). 2025 May 6;14(5):507. doi: 10.3390/biology14050507 (PMC12109162; doi:10.3390/biology14050507)

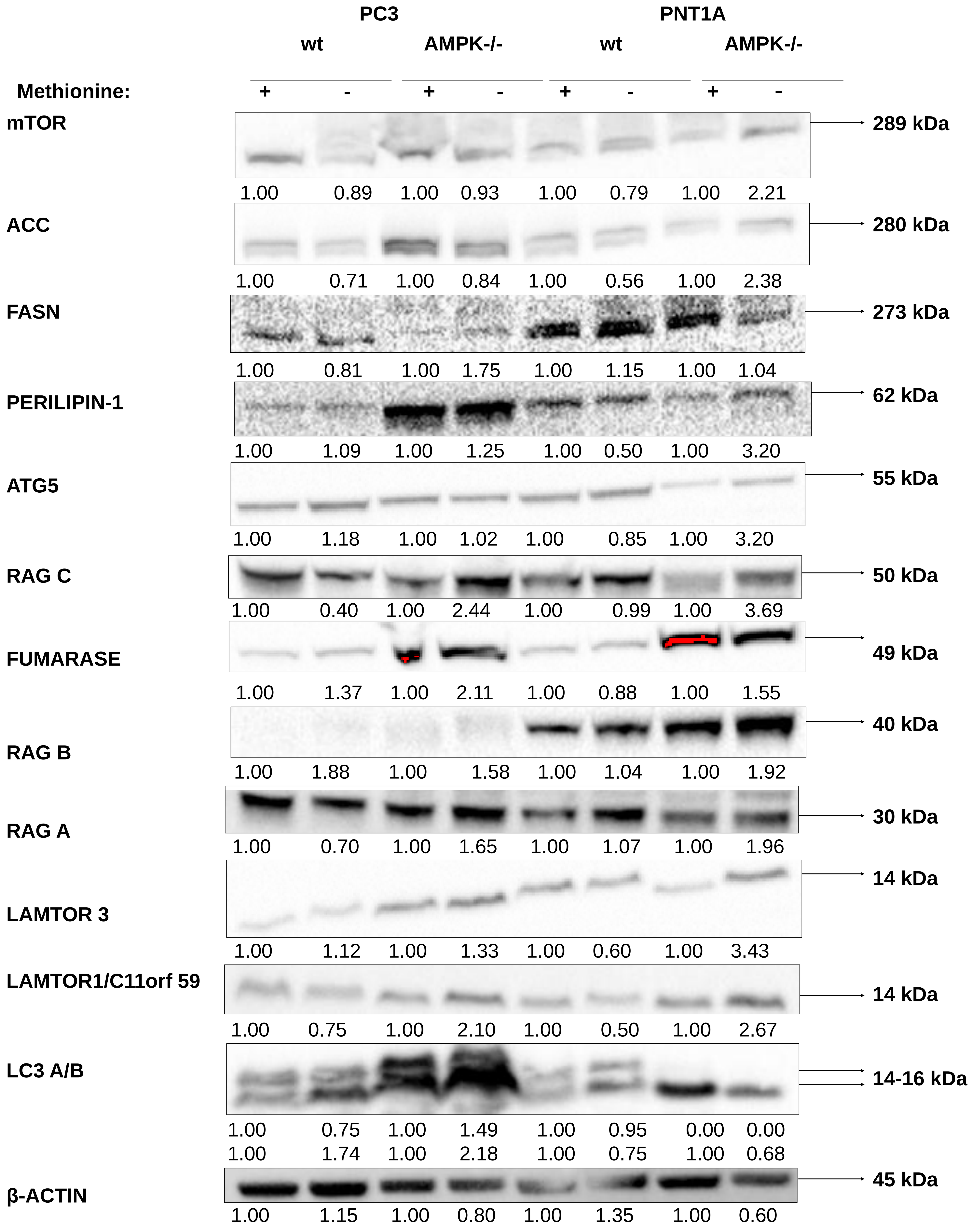
PC3
PNT1A
wt
AMPK-/-
wt
AMPK-/-
Methionine:
+ - + - + - + -
mTOR
1.00 0.89 1.00 0.93 1.00 0.79 1.00 2.21
289 kDa
ACC
1.00 0.71 1.00 0.84 1.00 0.56 1.00 2.38
280 kDa
FASN
1.00 0.81 1.00 1.75 1.00 1.15 1.00 1.04
273 kDa
PERILIPIN-1
1.00 1.09 1.00 1.25 1.00 0.50 1.00 3.20
62 kDa
ATG5
1.00 1.18 1.00 1.02 1.00 0.85 1.00 3.20
55 kDa
RAG C
1.00 0.40 1.00 2.44 1.00 0.99 1.00 3.69
50 kDa
FUMARASE
1.00 1.37 1.00 2.11 1.00 0.88 1.00 1.55
49 kDa
RAG B
1.00 1.88 1.00 1.58 1.00 1.04 1.00 1.92
40 kDa
RAG A
1.00 0.70 1.00 1.65 1.00 1.07 1.00 1.96
30 kDa
LAMTOR 3
1.00 1.12 1.00 1.33 1.00 0.60 1.00 3.43
14 kDa
LAMTOR1/C11orf 59
1.00 0.75 1.00 2.10 1.00 0.50 1.00 2.67
14 kDa
LC3 A/B
1.00 0.75 1.00 1.49 1.00 0.95 0.00 0.00
1.00 1.74 1.00 2.18 1.00 0.75 1.00 0.68
14-16 kDa
β-ACTIN
1.00 1.15 1.00 0.80 1.00 1.35 1.00 0.60
45 kDa

mTOR Antibody #2972

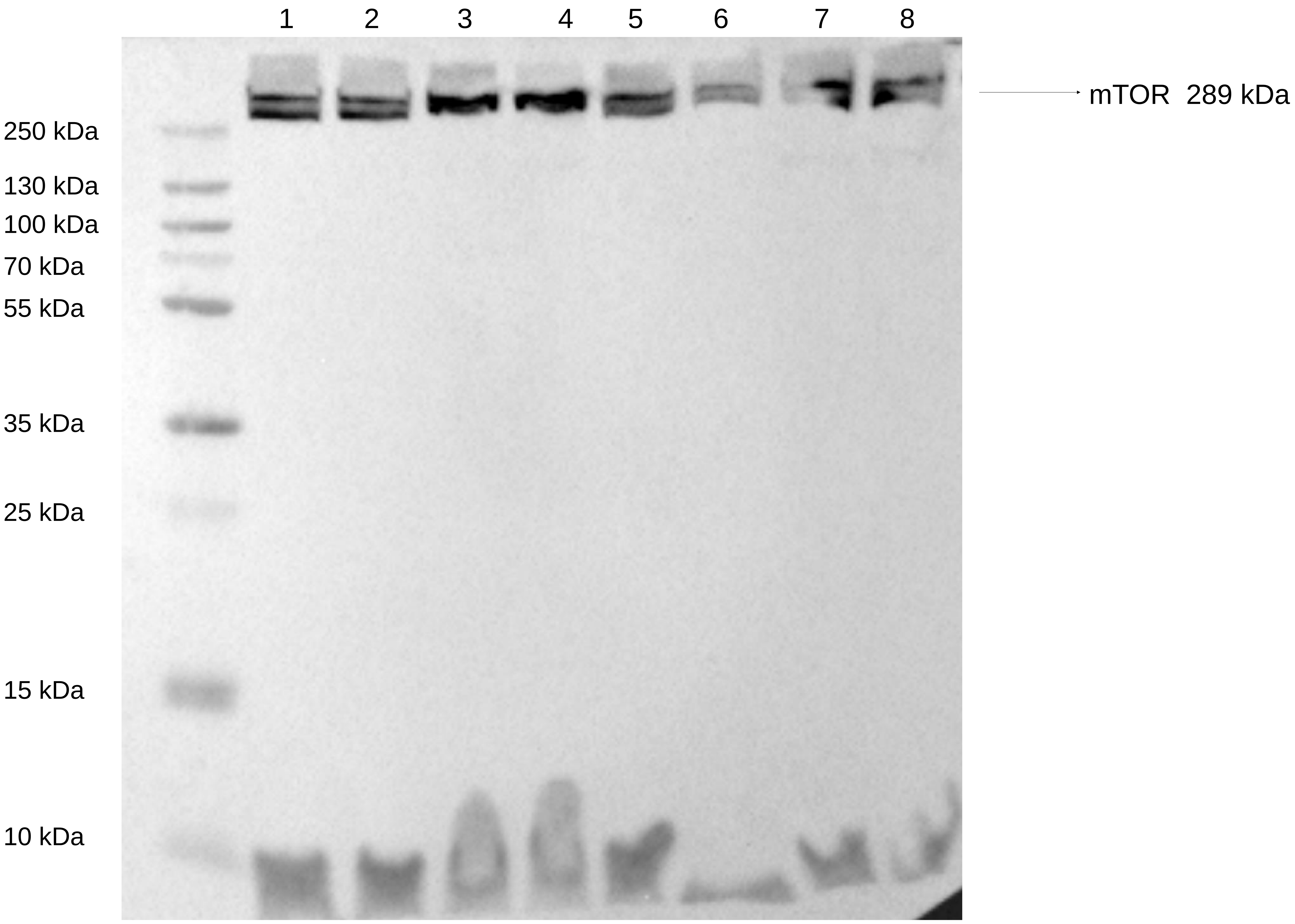

ACC-LC3 AB

ACC:Acetyl-CoA Carboxylase (C83B10) Rabbit mAb #3676

LC3 AB:LC3B Antibody #2775

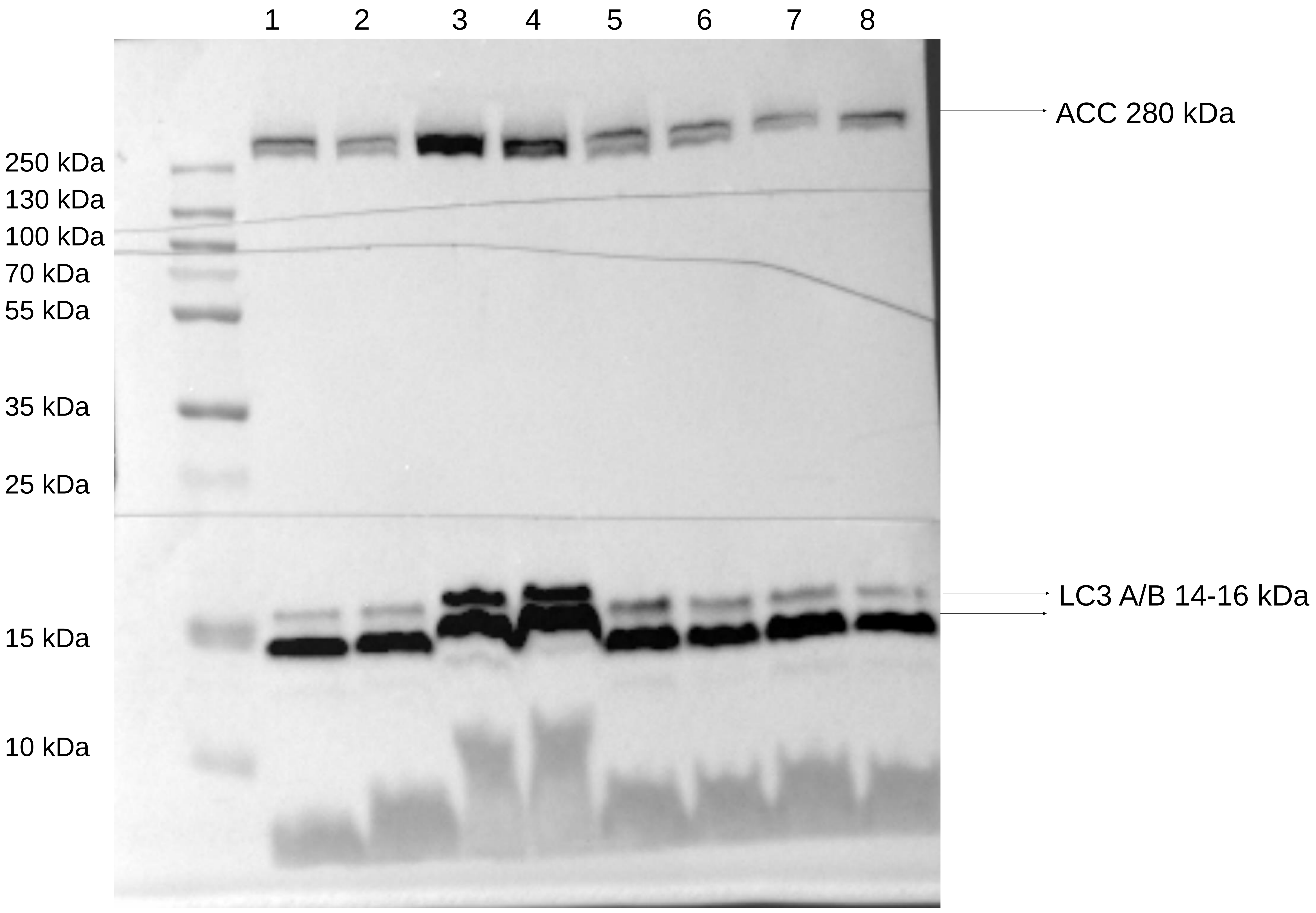

Fatty Acid Synthase (C20G5) Rabbit mAb #3180

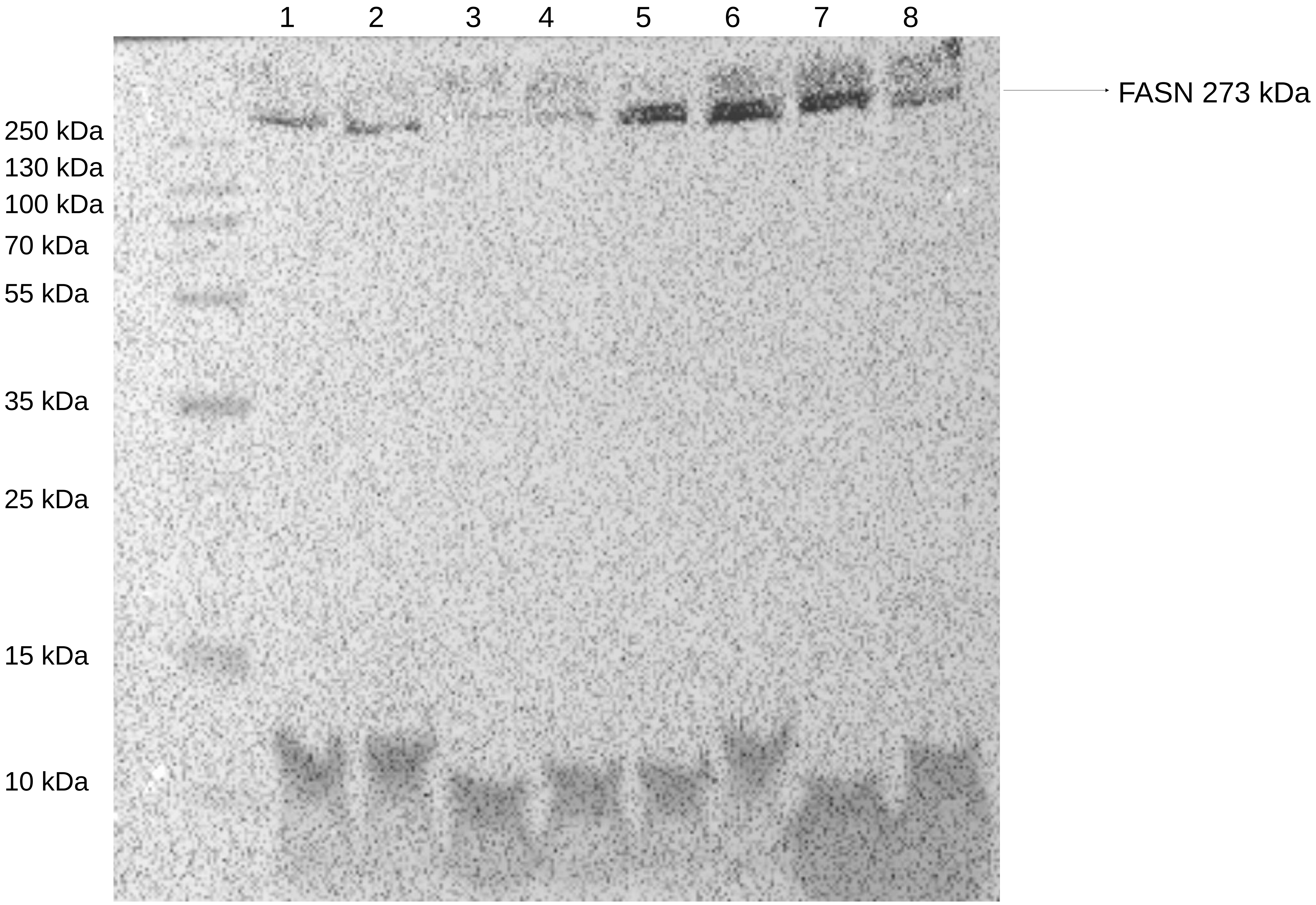

Perilipin-1 (D1D8) XP® Rabbit mAb #9349

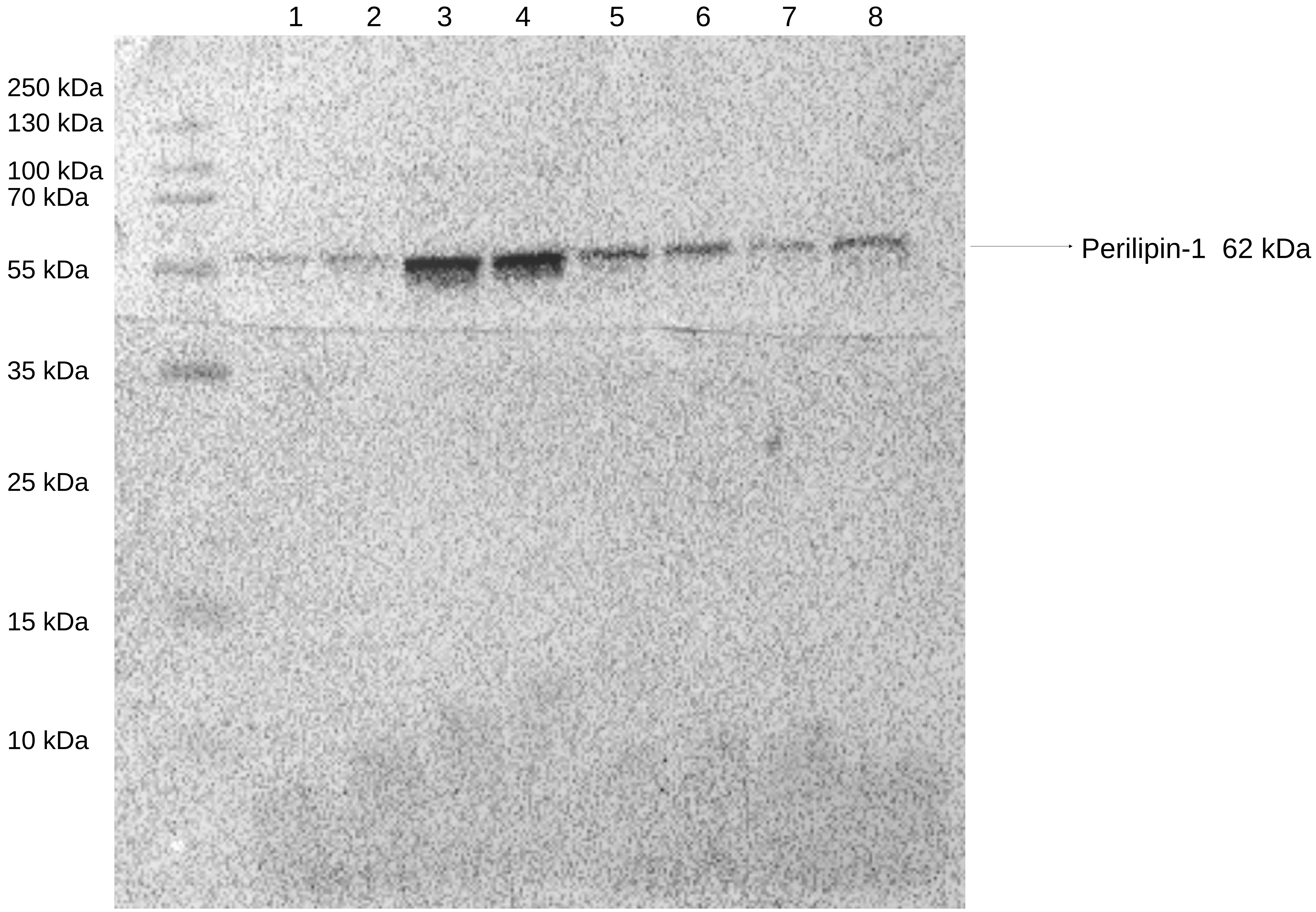

ATG5 Atg5 (D5F5U) Rabbit mAb #12994

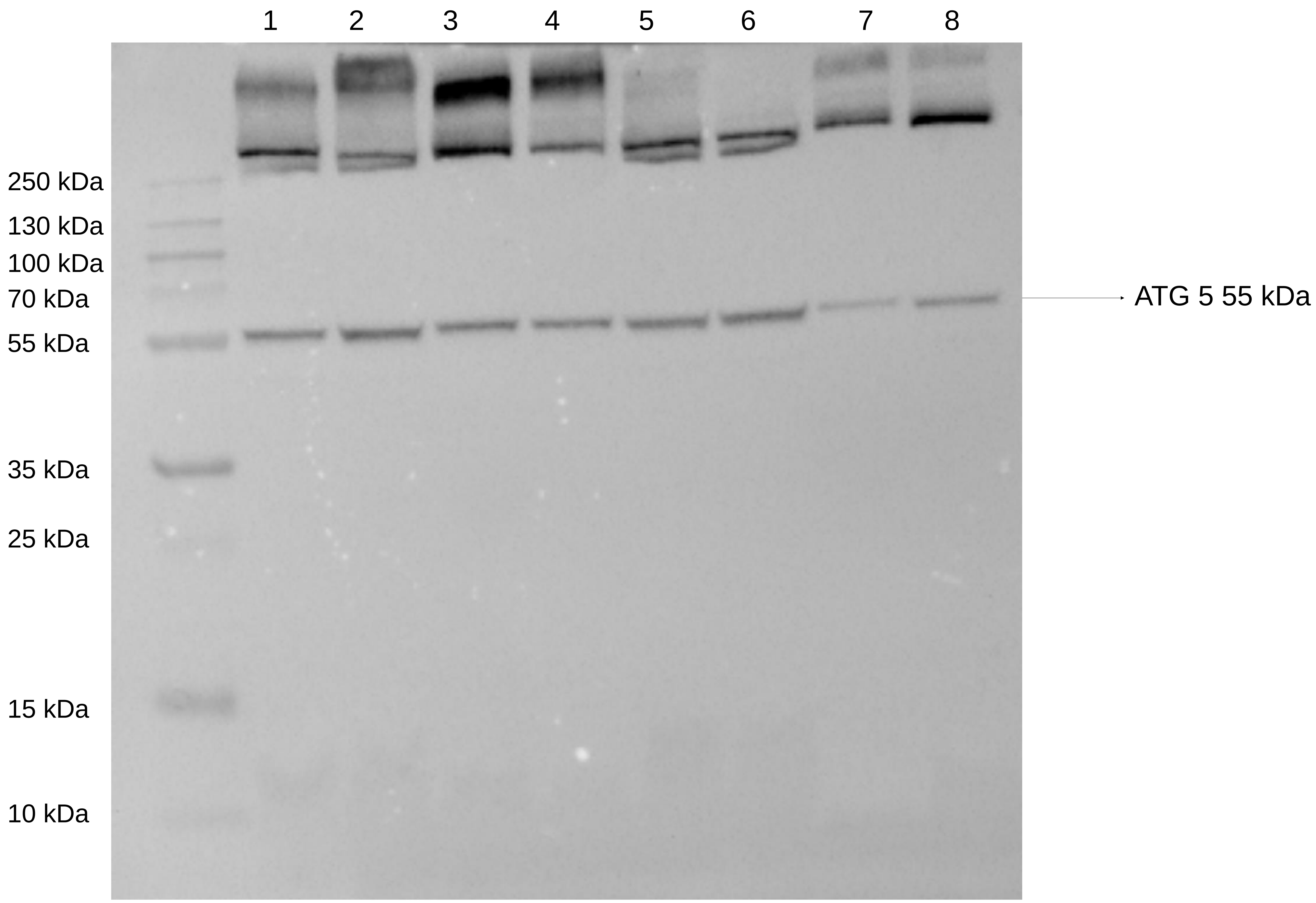

RagC (D8H5) Rabbit mAb #9480

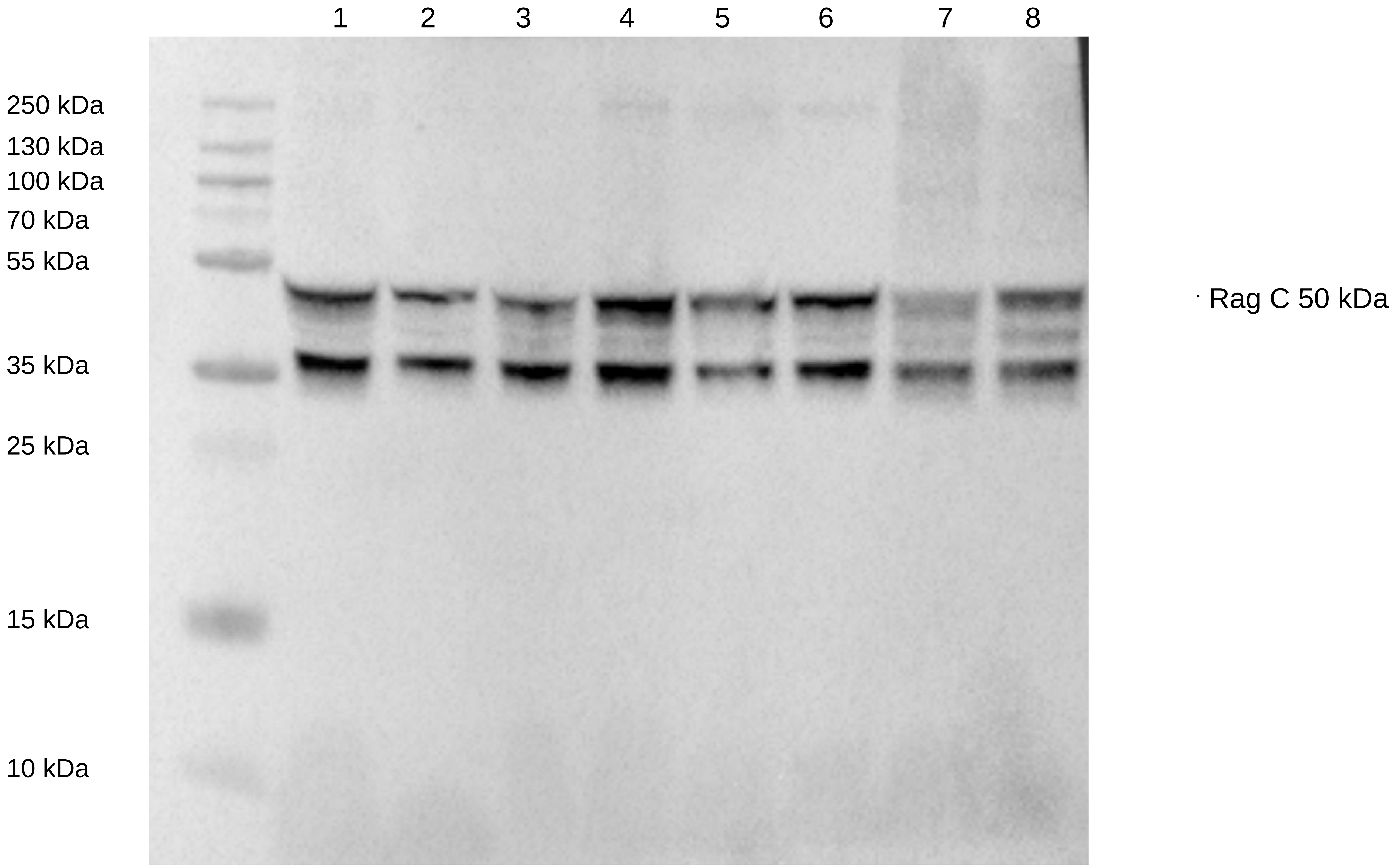

Fumarase (D9C5) Rabbit mAb #4567

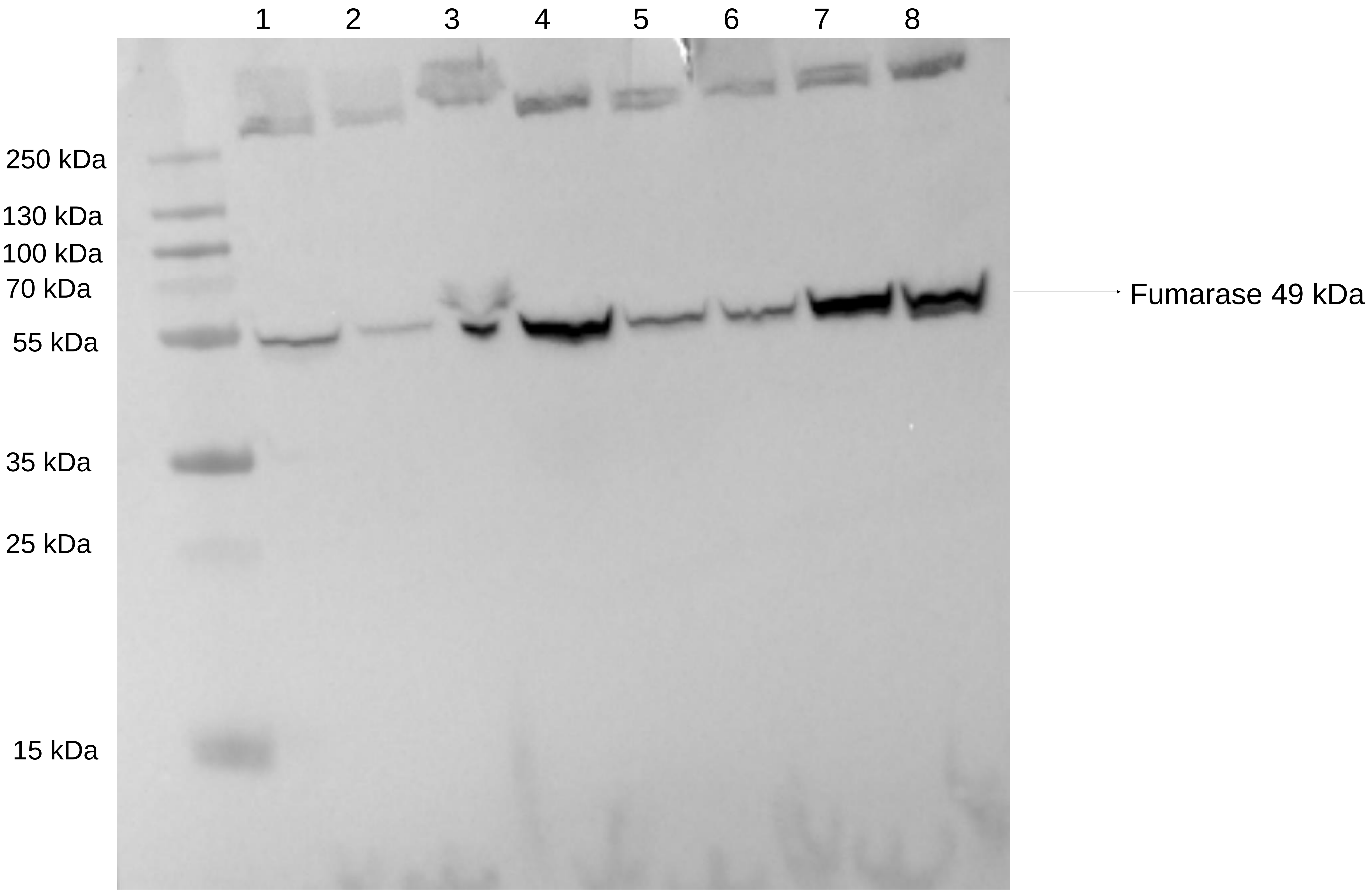

RagB (D18F3) Rabbit mAb #8150

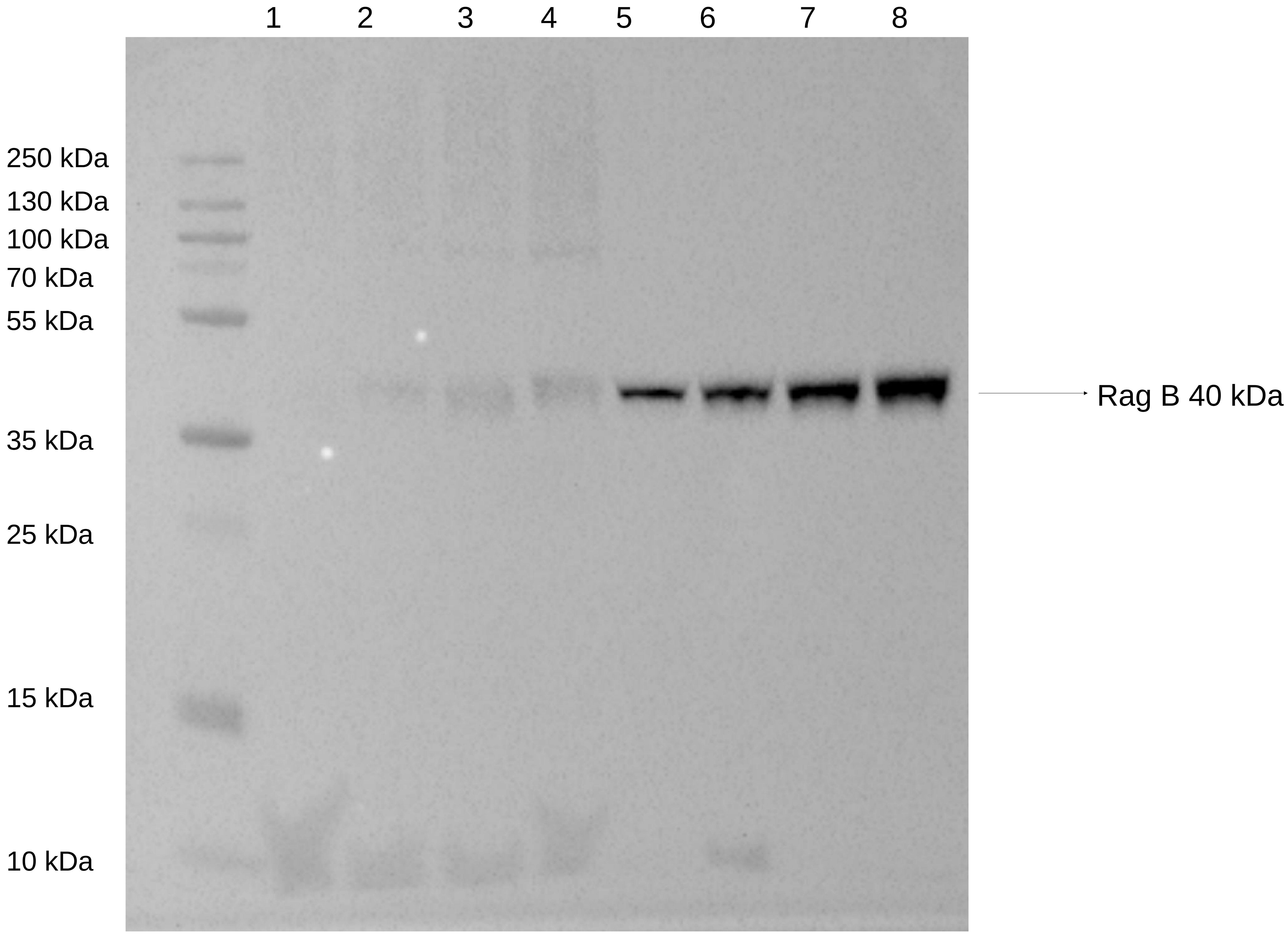

RagA (D8B5) Rabbit mAb #4357

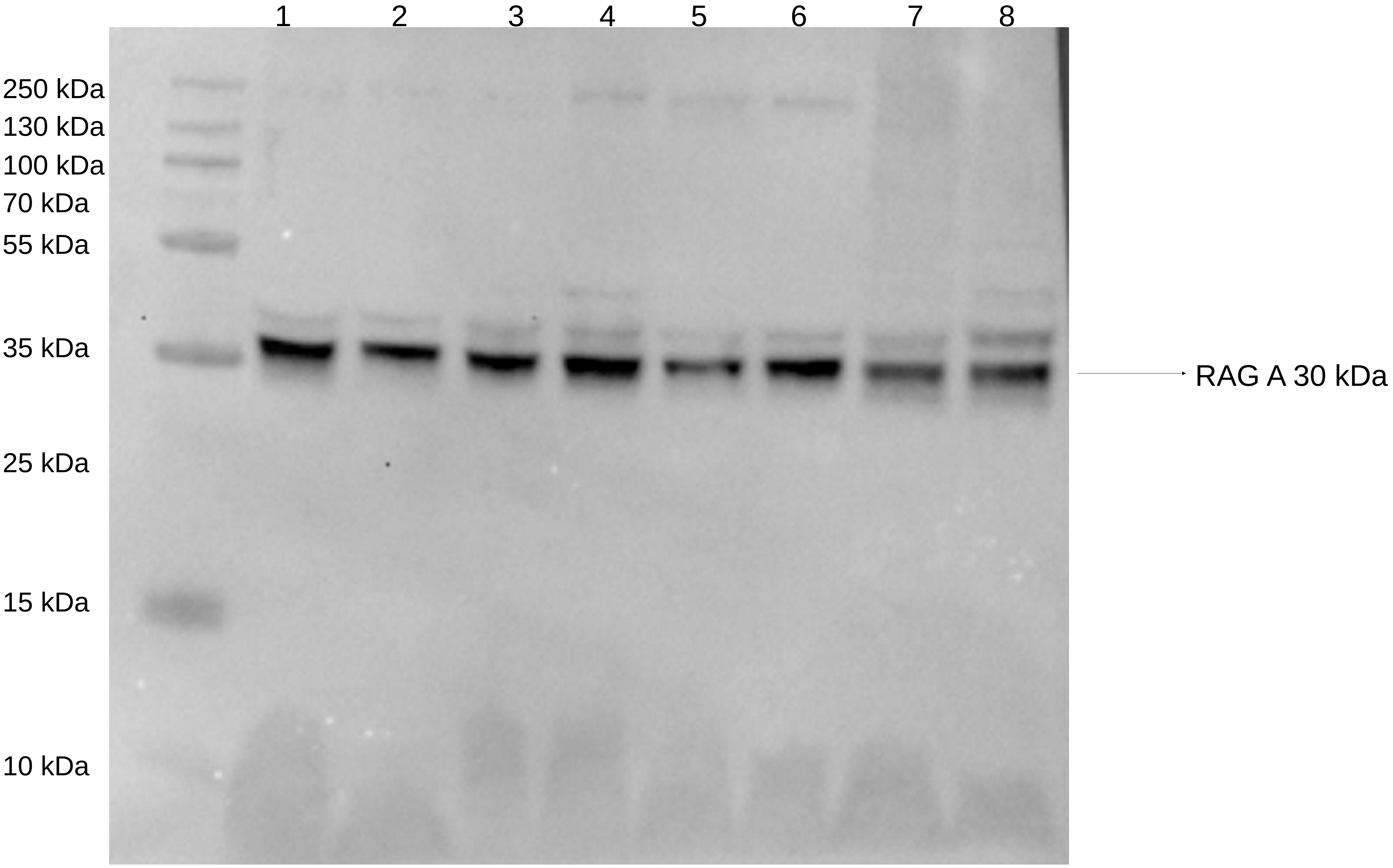

LAMTOR3/MAPKSP1 (D38G5) Rabbit mAb #8168

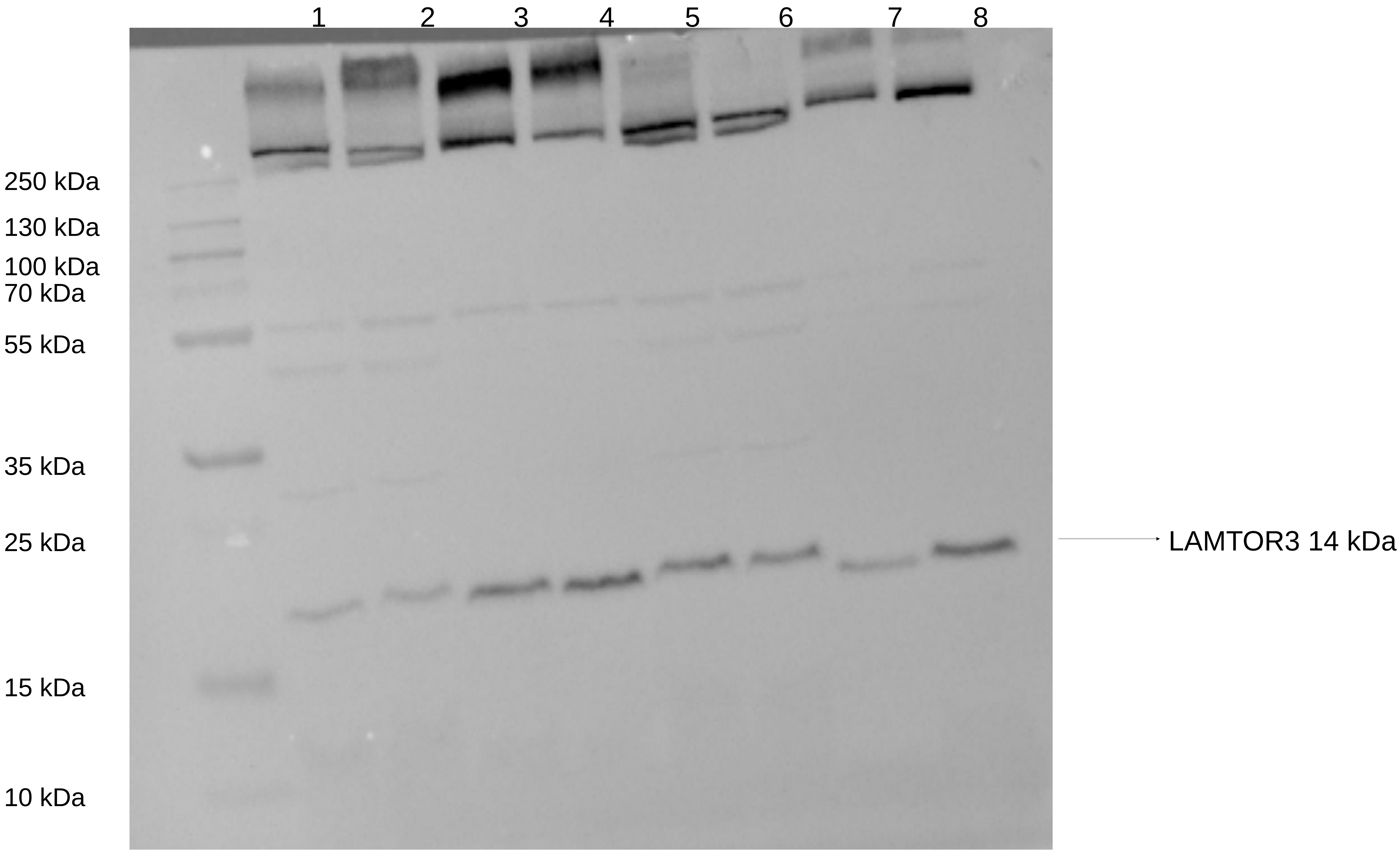

LAMTOR1/C11orf59 (D11H6) XP® Rabbit mAb #8975

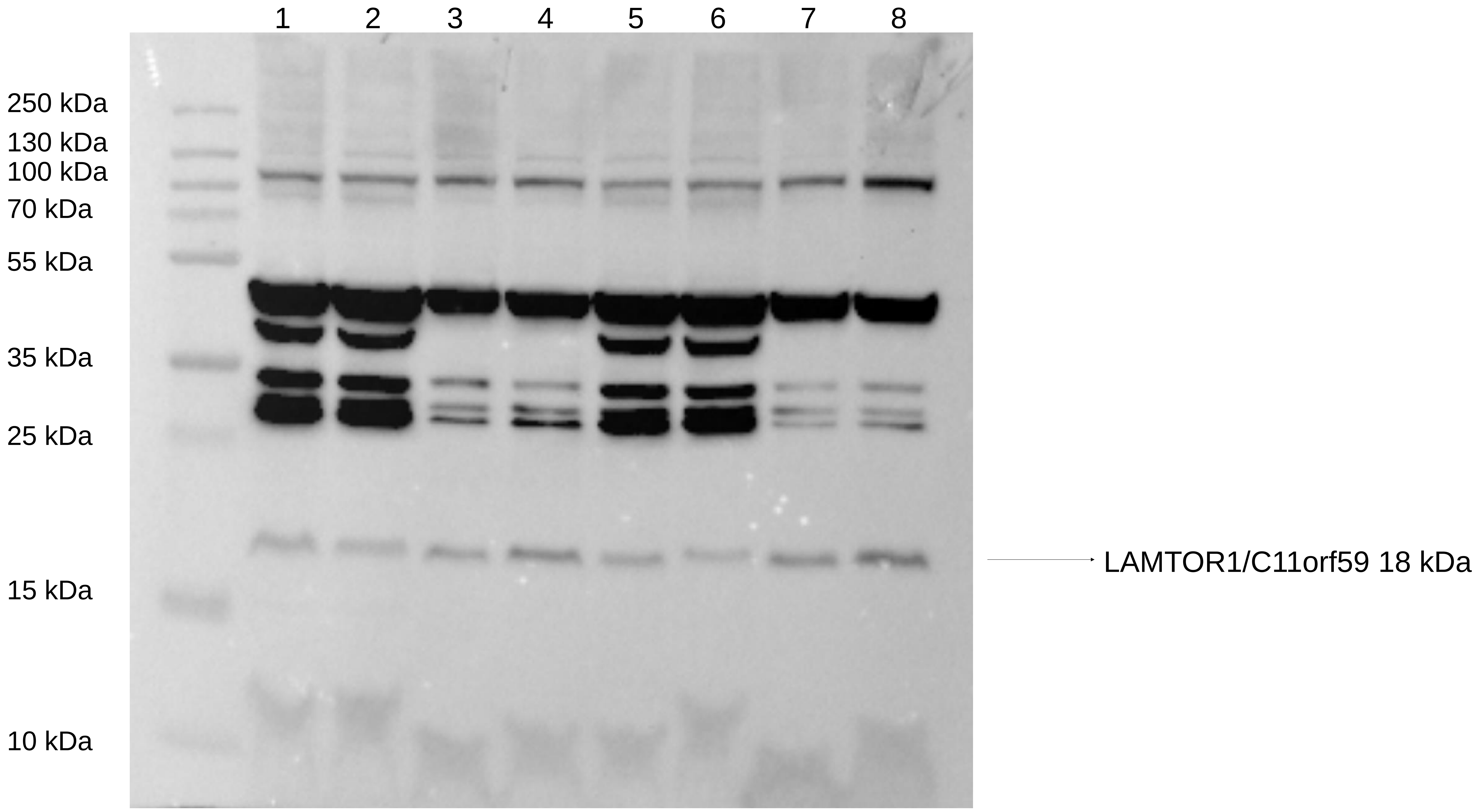

β-Actin Antibody #4967

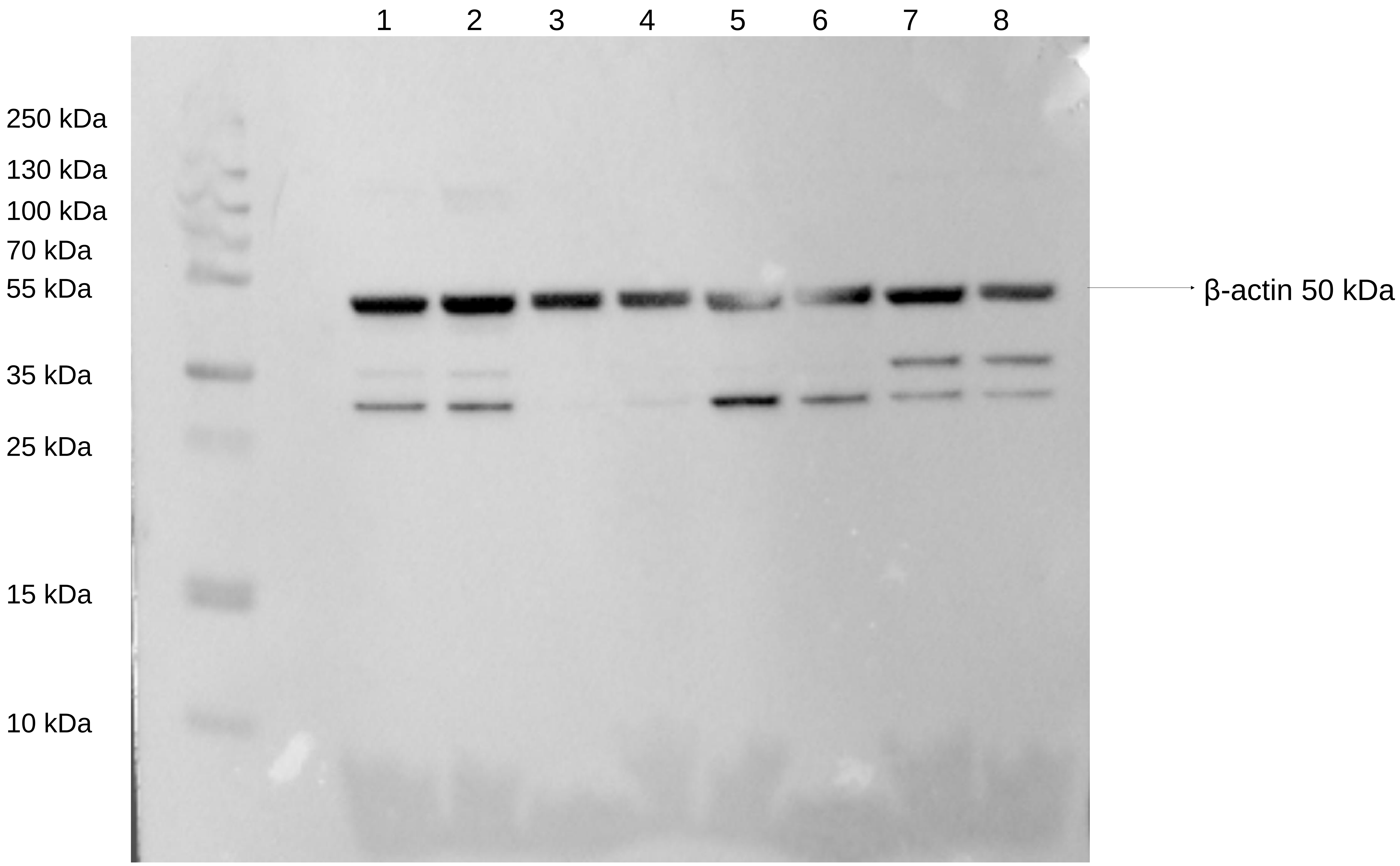

Supplement: Supplementary file 1 [file biology-14-00507-s001.zip › biology-3584047-supplementary Figures S4.pdf]
