# Supplementary material for: Targeting Methionine Metabolism Reveals AMPK-SAMTOR Signaling as a Therapeutic Vulnerability in Prostate Cancer
Source: Biology (Basel). 2025 May 6;14(5):507. doi: 10.3390/biology14050507 (PMC12109162; doi:10.3390/biology14050507)
Supplement: Supplementary file 1 [file biology-14-00507-s001.zip › biology-3584047-supplementary materials Figures S1-S3.pdf]

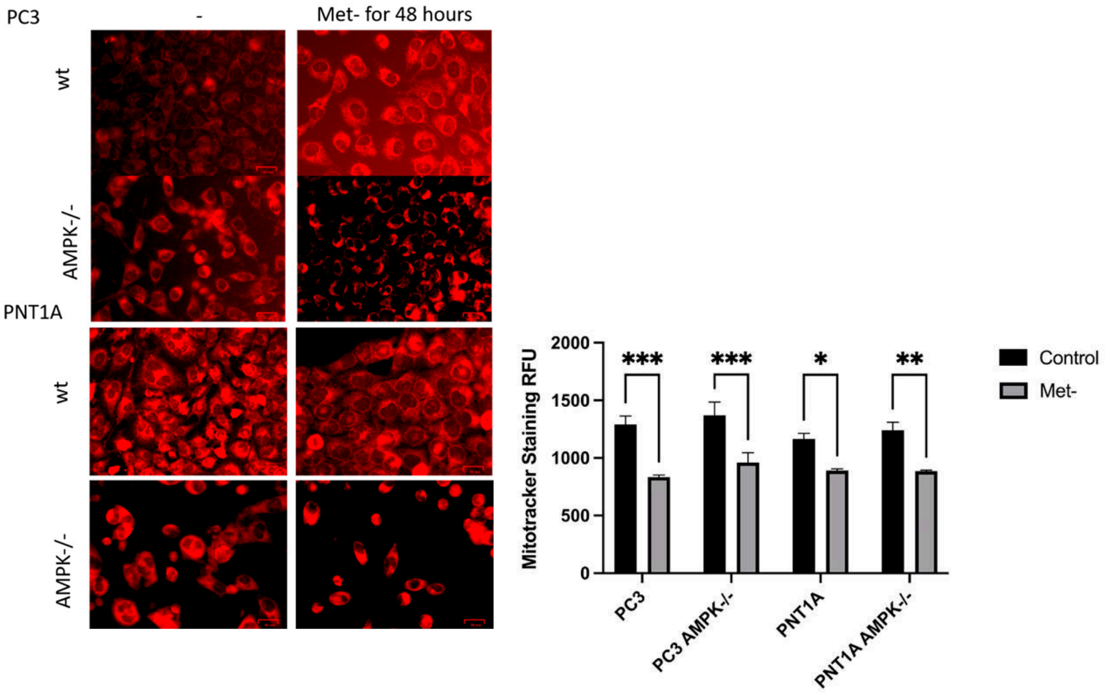


**Supplemental Figure S1.** Mitochondrial membrane potential decreases following 48-hour methionine deprivation, assessed by Mitotracker staining and fluorescence analysis. Cells were stained with Mitotracker dye (5 mg/mL) transferred from the stock solution and incubated for 15 minutes. Representative fluorescence microscopy images for each group are shown on the left. Fluorescence intensity was quantified using a plate reader at an excitation wavelength of 579 nm and an emission wavelength of 599 nm (right). The bar graph presents mean ± standard error from three independent experiments with two replicates each. Statistical analysis was performed using Sidak's multiple comparisons test following two-way ANOVA: *p = 0.109, **p = 0.0023, and ***p = 0.0004 (PC3 wt); ***p = 0.0009 (PC3 AMPK-/-). Scale bar: 25 μm.

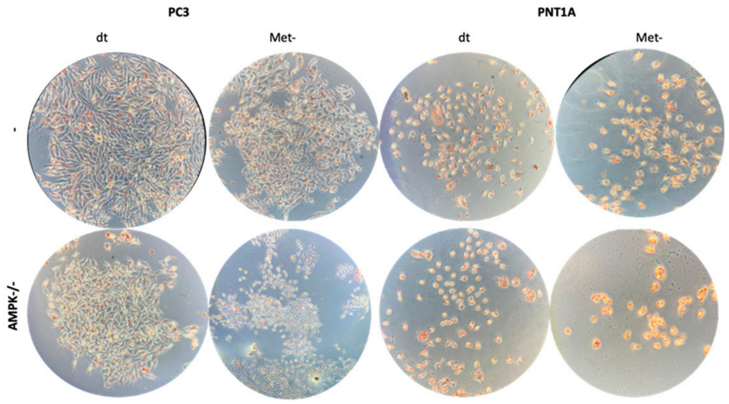


**Supplemental Figure S2.** Visualization of lipid droplet accumulation in PC3 and PNT1A wild-type and AMPK-/- cells by Oil Red O staining. Representative images show red-stained intracellular lipid droplets indicating interaction with Oil Red O dye. Images are representative of three independent experiments (n = 3).

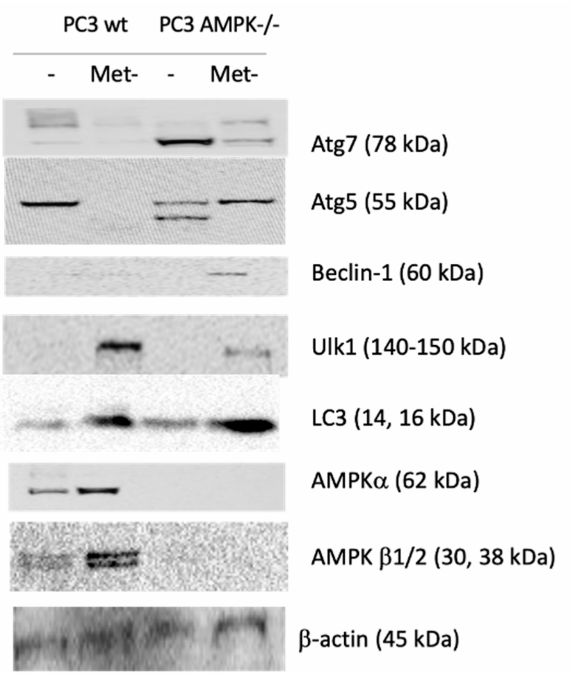


**Supplemental Figure S3.** Impact of methionine deprivation on autophagy-related signaling within the AMPK pathway in PC3 wild-type and AMPK-/- cells. Western blot analysis of autophagy-related signaling components in PC3 wild-type and AMPK-/- cells following 24 hours of methionine deprivation. For each sample, 30 μg of protein was loaded per lane. β-actin was used as a loading control. Data are representative of three independent experiments (n = 3).
